# Acknowledgement to Reviewers of International Journal of Molecular Sciences 2013

**DOI:** 10.3390/ijms15033444

**Published:** 2014-02-25

The editors of *International Journal of Molecular Sciences* would like to express their sincere gratitude to the following reviewers for assessing manuscripts in 2013:

Abass, Khaled
Abbott, Catherine A.
Abbott, Karen L.
Abbott, Wade
Abdelly, Chedly
Abdelmohsen, Kotb
Abe, Hiroshi
Abe, Jun-Ichi
Abe, Koji
Abe, S.
Aberger, Fritz
Aboshady, Ibrahim
Abou Neel, E.A.
Abou-Alfa, Ghassan
Abraham, W.R.
Abu-Soud, Husam M.
Achá, Darío
Ackermann, Paul W.
Ackland, K.
Acuña Castroviejo, Darío
Adachi, Souichi
Adams, Timothy E.
Addadi, Lia
Adkins, DeAnna L.
Adrián, Rodríguez-Burruezo
Adusumilli, Prasad S.
Afantitis, Antreas
Affourtit, Charles
Aft, Rebecca
Ageorges, Agnès
Aghaloo, Tara
Agoulnik, Irina
Aguilar, Daniel
Aguilera, José
Aguinaldo, Alicia
Aguirre, Windsor
Agulló-Ortuño, M. Teresa
Agustí, Javier
Agut, Montserrat
Agyei, Dominic
Ahammed, Golam Jalal
Ahmad, Aamir
Ahmed, Ibrar
Ahmed, Zubair
Ahn, Erin Eun-Young
Ahn, Suk-Kyun
Aizawa, Mineaki
Akerman, Ildem
Akhtar, Yasmin
Al Ghouleh, Imad
Alarcón, F.J.
Albano, Emanuele
Albrecht, Huguette
Alcolea Palafox, M.
Alexander, G.
Alexander, Henry
Alexeyev, M.
Alexiou, Christoph
Ali, Mubarak
Aliaga-Alcalde, Nuria
Alison, Malcolm R.
Aliverti, Alessandro
Allan, Andrew C.
Allard, Emmanuel
Allen, Randy
Alliston, Tamara
Allsopp, R.
Almerico, Anna Maria
Almqvist, Sofia
Altieri, Fabio
Altstädt, Volker
Álvarez-Dolado, Manuel
Alvarez, Ezequiel
Alvarez, J.B.
Amâncio, S.
Amarasekara, Ananda S.
Amasheh, Salah
Ambrosini, Grazia
Ambs, Stefan
Amelia, Matteo
Amendola, Vincenzo
Amzel, L. Mario
Anacker, Christoph
Anastasi, Emanuela
Ancans, Janis
Anders, Carey K.
Andersen, Otto
Anderson, Matthew L.
Anderson, Travis M.
Andersson, My S.
Ando, H.
Andrade, Fernanda
Andrade, Paula Branquinho
Andreev, Oleg A.
Andrisano, Vincenza
Aneja, Ritu
Angele, Peter
Angelova, Angelina
Anglani, Franca
Angulo, Javier
Anker, Jeffrey N.
Antohe, Felicia
Antonio, Carla
Aono, Toshihiro
Apte, Udayan
Aquila, saveria
Aquino, Rita
Arai, Ryoichi
Araki, Toshiyuki
Aranda, Agustín
Aránega, Amelia E.
Aransay, Ana M.
Arany, E.
Araújo Pereira Coutinho, João Manuel Costa
Aravalli, Raj
Arbona, Vicent
Arceci, Robert J.
Archer, Trevor
Arculeo, Marco
Arena, Mario Eduardo
Arens, Ramon
Argawa, Ankit
Ariga, Katsuhiko
Arisz, S.A.
Armamento-Villareal, Reina
Armentero, Marie-Therese
Armstrong, Alicia Y.
Arnason, John Thor
Arnesen, Thomas
Arnold, Julia T.
Aroca, Ricardo
Aronheim, Ami
Arques, A.
Arrighi, S.
Artavanis-Tsakonas, Spyros
Arthur, Michel
Arya, Dev
Asaga, Sota
Aschberger, Karin
Ashida, Hitoshi
Ashida, Kanae
Ashley, Neil
Asiegbu, Fred O.
Aslan, Kadir
Assimakopoulos, Stelios
Ata, Athar
Atkinson, Nicky J.
Atrens, Andrej
Attia, Mohammed
Atwood, Craig S.
Atzler, Dorothee
Audet, Patrick
Aukema, Harold M.
Ault, Bruce S.
Austriaco, Nicanor
Averous, Julien
Avery, Mitchell A.
Avesani, Linda
Ávila-Flores, Antonia
Avila, Carlos
Avila, Matias A.
Avruch, Joseph
Ayala-Sanmartin, Jesus
Ayasolla, Kameshwar R.
Ayyalusamy, Ramamoorthy
Azevedo, L.
Azevedo, Mauro F.
Azevedo, Ricardo B.
Aziz, Syed
Azmi, Asfar S.
Azuma, Akifumi
Azuma, Hideki
Baba, Hideo
Baba, K.
Babitzke, Paul
Bach, Horacio
Bachelerie, Françoise
Bachovchin, William W.
Bachschmid, Markus M.
Badea, Ildiko
Bae, Hae-Rahn
Bae, Hyeun-Jong
Bae, Yoe-Sik
Baek, Seung-Soo
Baek, Suk-Hwan
Baena-González, Elena
Bagavandoss, P.
Bagella, Luigi
Baginski, Maciej
Baici, Antonio
Baier-Bitterlich, Gabriele
Bailey, Susan M.
Bain, Lisa J.
Bais, Harsh P.
Bajwa, Amandeep
Bak, Ann Mosegaard
Baker, Andrew
Baker, Mark D.
Bakkenist, Chris
Balázs, Barna
Balcells, Mercedes
Baldanzi, Gianluca
Baleja, Jim
Balemba, Onesmo B.
Balestrazzi, Alma
Balius, Trent
Ballhorn, Daniel J.
Ballizany, Wouter L.
Balmas, Virgilio
Bamdad, Cynthia
Banchio, Erika
Banerjee, Partha
Banga, J. Paul
Baniahmad, Aria
Bansal, Seema
Banu, Jameela
Bao-Sheng, Li
Bao, Yongping
Bao, Yuping
Baranzini, Sergio E.
Barassi, Alessandra
Barber, Domingo F.
Barderas, Maria G.
Bardoni, Barbara
Bardor, Muriel
Barnett, Matthew P.G.
Baron, J.M.
Baron, Riccardo
Barrett-Jolley, Richard
Barriere, Yves
Barth Jr., Richard J.
Bartlett, Madelaine E.
Bartolome, Juan
Bartoloni, Giovanni
Barz, Bogdan
Basini, Giuseppina
Baskoutas, Sotirios
Bast, A.
Bastiat, Guillaume
Basu, Partha
Bathgate, R.A.D.
Batlle, Eduard
Battaglia, Eric
Bauer, Johann
Bauer, Mario
Baulin, Vladimir A.
Baum, Oliver
Baumgärtel, Viola
Baureder, Michael
Bautista-Gallego, J.
Bavaresco, Luigi
Bay, Boon-Huat
Bayat, Abolfazl
Beadle, G.
Beck, Melinda A.
Becker, Norbert
Becker, Therese M.
Bednarek, Paweł
Beemon, Karen
Bégin, André
Behr, Matthias
Bejai, Sarosh
Bekaert, Bram
Belkacemi, Louiza
Bella, Federico
Bellini, Tommaso
Bellón, Teresa
Bellucci, Devis
Bellucci, Michele
Belobrajdic, Damien P.
Beloglazkina, E.K.
Bem, Reinout Alexander
Benaissa, Azzedine
Benavente, Elena
Bender, Nicole
Benito, Juan M.
Benjamim, Claudia Farias
Benner, S.A.
Benoit, Joshua
Bentley, T.W.
Bentsen, H.
Benzeroual, Kenza E.
Berezin, Mikhail Y.
Bergamaschi, Enrico
Bergan, Raymond C.
Berger, Philipp
Bergheim, Ina
Bergmann, Pierre
Berkel, Gary J. Van
Berman, Nancy E.J.
Bernabeu, Carmelo
Bernad, A.
Bernasconi, P.
Bernstein, Hans-Gert
Bernstein, Peter
Berrebi, Patrick
Berridge, Michael V.
Berrocal-Lobo, Marta
Berruti, Giovanna
Bert, Theresa M.
Bertoli, Alessandro
Bessega, C.
Betley, Theodore A.
Betterle, Corrado
Bettington, Mark
Betzel, Christian
Beverly, Levi J.
Bezdetnaya, Lina
Bezverkhyy, Igor
Bhatia, Sujata
Bhattacharjya, Surajit
Bhushan, Shashi
Biamonti, Giuseppe
Biedermannová, Lada
Biel, Martin
Bietti, Massimo
Bignotti, E.
Bilbao, Cristina
Billard, Isabelle
Bills, Gerald F.
Billuart, P.
Binétruy, Bernard
Bingle, Colin
Birbilis, Nick
Birgitta Langhammer, Birgitta
Birol, İnanç
Birringer, Marc
Bisaglia, Marco
Bishop, Anthony C.
Biswas, Pradip K.
Bitonti, Maria Beatrice
Bjerregaard-Andersen, Morten
Bjorkling, Fredrik
Blair, Harry
Blaise, G.A.
Blanc, Christophe
Blanchard, Frédéric
Blanco, Gloria
Blanco, José L. Jiménez
Blasiak, Janusz
Bleich, Andre
Blenau, Wolgang
Blennerhassett, Michael
Blight, Andrew
Bloch, Wilhelm
Block, Stephan
Bloise, E.
Board, Philip
Bobola, Nicoletta
Bocchinfuso, Gianfranco
Boehmer, Jamie
Boerma, Marjan
Bogan, Jonathan S.
Bogel-Łukasik, Rafal
Bohm, L.
Boine, Barbara
Boix, Ester
Boles, Blaise R.
Bolognesi, Claudia
Bommer, Ulrich-Axel
Bonavida, Benjamin
Bonci, Desiree
Bonetti, Bruno
Bonifácio, Vasco
Bono, Petri
Booth, David
Booth, Martin J.
Borlee, Brad
Borsani, Elisa
Boschi, Donatella
Bosnjak, Zeljko
Bothner, Brian
Botta, Luigi
Bottle, Steve
Boudjema, K.
Boudreau, Luc
Boumpas, Dimitrios
Bourque, Stéphane
Boutin, Jean
Bouvet, Michael
Bouwmeester, Harro J.
Bové, J.
Bowen, Phyllis
Bowyer, Michael C.
Bozue, Joel
Bozzelli, Joseph W.
Bozzini, Sabrina
Braam, Wiebe
Bracken, Cameron P.
Brackman, Gilles
Bradford, Barry
Bradley, Walter G.
Bradshaw, Patrick C.
Braicu, E.I.
Braissant, O.
Brait, Mariana
Brancati, Francesco
Brandel, Daniel
Brandon, Amanda
Brantestam, Agnese Kolodinska
Braun, Latoya Jones
Braun, Ruedi K.
Braun, Thomas
Bravenboer, Nathalie
Bray, Dennis
Brazel, Christopher S.
Breinbauer, Rolf
Breitenbach, Michael
Breitkopf-Heinlein, Katja
Breitwieser, Wolfgang
Brender, Jeffrey R.
Brenndörfer, Erwin
Bretagne, S.
Brew, B.J.
Brewer, Gregory J.
Brewer, Philip B.
Briner, Wayne
Briones, Ana M.
Brito, Maria Alexandra
Britto, Dev T.
Broer, I.
Brooks, Donald E.
Brosens, J.J.
Brotman, Yariv
Brown, Eric L.
Brown, Lindsay
Brown, Mark A.
Brubaker, Patricia L.
Bruce, Kimberley D.
Brugts, J.J.
Bruinink, Arie
Brunelle, Alain
Brunetti, Antonio
Brunetti, Gennaro
Brunham, Liam R.
Bruning, Bas
Brunklaus, Günther
Brunsveld, Luc
Bruserud, Øystein
Brusslan, Judy A.
Bucher, Denis
Buchete, Nicolae-Viorel
Buckanovich, Ronald
Buckel, Wolfgang
Budunova, Irina
Buechler, Christa
Bui, Bernadette Tse Sum
Bulut, Mustafa
Buonaccorsi, Vincent P.
Burdette, Joanna E.
Burger, Maximilian
Burgess, Karl E.V.
Burlak, Christopher
Burritt, David
Busch, Christian
Buse, Helen Y.
Bussolati, Benedetta
Bussolino, Federico
Bustin, Stephen A.
Butler, Deborah
Butler, Ivan Maureira
Butnariu, Monica V
Büttner, A.
Bye, Nicole
Byler, Timothy
Byrne, Bernadette
Byrne, Robert A.
Cabarcas, Stephanie
Cabello, Gérard
Cabot, Catalina
Cabral-Romero, Claudio
Cabrera, Carmen
Cai, Weili
Caimi, G.
Cakouros, Dimitrios
Calder, Philip
Calder, Virginia
Caliceti, Paolo
Calligari, Sebastián D.
Calvo, Florent
Calvo, José Luis
Camacho-Cristóbal, Juan J.
Camara, Amadou K.S.
Câmara, Niels Olsen Saraiva
Camins, Antoni
Campagna, Shawn R.
Campbell, Christopher
Campbell, Colin
Campbell, Ewan
Campbell, Fiona M.
Campbell, Sharon
Camps, Jordi
Canevari, Silvana
Canny, Geraldine
Canonico, Marianne
Cao, Dengfeng
Cao, Fang
Cao, Jake D.
Cao, Pengxiu
Capasso, Raffaele
Capell, Teresa
Capitanio, Nazzareno
Cappitelli, Francesca
Capra, Valérie
Carabelli, V.
Caraglia, Michele
Cardinali, Daniel P.
Cardon, Ludwig
Cardoso, Claudia R.L.
Carella, Angelo M.
Carlomagno, G.
Carlyle, James R.
Carnero, Amancio
Carnevali, O.
Caron-Debarle, Martine
Carpaneto, Armando
Carpéné, Christian
Carrano, Carl J.
Carrier, Danielle Julie
Carrubba, Alessandra
Carta, Fabrizio
Carter, Dee
Cartmill, Donita L.
Caruso, Gerardo
Carvajal, M.
Carvajal, Richard D.
Carvalho, Joana
Casarini, Dulce
Casas, Ana M.
Casorelli, Ida
Cassagnau, P.
Castanho, Miguel
Castrén, Maija L.
Castresana, Javier
Castro, Rui E.M.
Cataldi, Amelia
Catara, Vittoria
Catling, Andrew D.
Cavallini, Massimiliano
Cavicchi, Kevin
Cawthon, Richard M.
Cayetanot, Florence
Cecconi, Ciro
Ceci, Luigi R.
Ceder, Yvonne
Celenza, Giuseppe
Cellek, S.
Celli, Anna
Cerca, Nuno
Ceresini, Graziano
Ceriotti, Aldo
Cesano, Alessandra
Cesari, A.
Cha, Ji-Young
Chabi, Béatrice
Chacińska, Agnieszka
Chadee, D.N.
Chakraborty, Raja
Challet, Etienne
Chalmers, Jeffrey J.
Chami, Mounia
Chan, Anthony S.L.
Chan, Cheng
Chan, Cheong Xin
Chan, Elsa C.
Chan, King Ming
Chan, Kun-Ming
Chandler, John W.
Chang, Chin-Sung
Chang, Fang-Rong
Chang, Hai-Chou
Chang, Jeong Ho
Chang, Li-Ching
Chang, Long-Sen
Chang, Ming-Chung
Chang, Raymond Chuen-Chung
Chang, Wen-Wei
Chang, Yu-Ming
Chapel, Alain
Chapman, C.A.
Chapman, James
Chappell, Mark C.
Chari, Divya M.
Charlier, Daniel
Charra, Fabrice
Chaudhary, Kunal
Chaudière, Jean
Chauhan, Vinita
Chaung, Hso-Chi
Chaurand, Pierre
Chekanova, Julia A.
Chellaiah, Meenakshi
Chelli, Riccardo
Chen, Bernadette
Chen, Chieh-Fu
Chen, Chu-Huang
Chen, Chung-Ming
Chen, Chung-Yi
Chen, Chunjung
Chen, Feng
Chen, Grace Y.
Chen, Gwo-Shing
Chen, Huaping
Chen, Jem-Kun
Chen, Jiann-Chu
Chen, Jiezhong
Chen, Jih-Jung
Chen, Jiun-Rong
Chen, Mingyi
Chen, Pin-Yuan
Chen, Rui
Chen, Shuei-Yuan
Chen, Tso-Hsiao
Chen, Yen-Hao
Chen, Yiwang
Chen, Yongsheng
Chen, Yu
Chen, Yuan
Chen, Yung-hsiang
Chen, Zhiguo
Chenais, Benoit
Cheng, Hai-Ying M.
Cheng, Jason Chia-hsein
Cheng, Jing-Jy
Cheng, Ming
Cheng, Nikki
Cheng, Zhenyu
Cheng, Ziyong
Cheong, Jae-Ho
Cheong, Yong Hwa
Chern, Ming-Kai
Cherng, Jong-Yuh
Chernin, L.
Cheung, Any
Cheung, David
Cheung, Wing-Hoi
Chi-Shing Cho, William
Chi, Qijin
Chiang, Meng-Tsan
Chiang, Tai-an
Chiaravalli, Anna Maria
Chiarello, Gennaro
Chiba, Mitsuru
Chichger, Havovi
Chiellinia, Emo
Chien, A.J.
Chiorino, Giovanna
Chirumbolo, Salvatore
Chitnis, Nilesh
Cho, Chi Hin
Cho, Dong-Woo
Cho, Nam Hoon
Cho, Wan-Seob
Choi, Byung Tae
Choi, Donghoon
Choi, Hyoung Jin
Choi, Il-Whan
Choi, In-Young
Choi, nyeong
Choi, Sang Tae
Choi, Seok Ki
Choi, Soon Gang
Choi, Wahn Soo
Choi, Yoon-La
Chomilier, Jacques
Chong, Kang
Chopp, Michael
Chopra, Mridula
Chopra, Surinder
Chorianopoulos, Nikos G.
Chou, Danny Hung-Chieh
Chouaib, Salem
Choudhary, Sikander Pal
Chow, Edward Kai-Hua
Chowdhury, Kamal
Christensen, Knud Villy
Christians, Elisabeth
Christos, Georgiou
Christov, Christo Z.
Chruszcz, Maksymilian
Chrzanowski, Łukasz
Chrzanowski, Wojciech
Chu, Ching-Liang
Chua, C.K.
Chuang, Yung-Jen
Chun, Jerold
Chung, Donghwa
Chung, Theodore D.
Churchill, David G.
Chuu, Chih-Pin
Chyan, Chia-Lin
Ciafrè, Silvia Anna
Cianci, Rosario
Ciaudo, Constance
Ciccotosto, Joe
Cicero, Arrigo F.G.
Cicero, Daniel O.
Cieśla, Łukasz
Cifelli, Mario
Cignarella, Andrea
Cinar, Bekir
Cirstea, Ion C.
Citterio, D.R.
Clarissa, Maroon-Lango
Clark, Catharine G.
Clausen, Frederik Banch
Clawson, Gary A.
Clegg, P.D.
Clop, Alex
Clouston, Andrew D.
Coate, Jeremy E.
Coccetti, Paola
Coenye, Tom
Cohen-Gonsaud, Martin
Coiras, Mayte
Colacino, Joseph M.
Colbert, Robert A.
Colby, David A.
Coleman, Anthony W.
Coleman, William B.
Coll, Josep
Collery, Ross F.
Collina, Simona
Collingridge, Graham L.
Collins, Mary
Collins, Maurice N.
Columbano, Amedeo
Comes-Franchini, Mauro
Comesse, Sébastien
Condorelli, Gianluigi
Condorelli, Momina
Contestabile, Antonio
Cook, Atlanta G.
Corbett, Anita H.
Corcoran, Nm
Cordero-Otero, Ricardo
Cordero, Gustavo
Cornall, Lauren
Cornett, Shannon
Corominas, M.
Correa, Fernando
Corrigan, Frances
Corsi, Fabio
Cortie, Michael B.
Cortright, Ronald N.
Corydon, T.J.
Cos, S.
Costa, Júlia
Costa, Valerio
Costagliola, Ciro
Costantini, Susan
Costantino, Luca
Côté, Jean-François
Cotsarelis, George
Cottet, Martin
Coulomb, Roger
Coulombe, Roger A.
Couture, Réjean
Coyle, Dennis E.
Cragg, Peter J.
Crea, Francesco
Cremer, Dieter
Crujeiras, Ana B.
Cruz, L.
Crynen, Gogce
Csősz, Éva
Cubero, Jaime
Cucchiarini, Magali
Cuendet, Michel
Cui, Daxiang
Cuk, Katarina
Culig, Zoran
Culot, Maxime
Cummings, Jeffrey
Curnow, Owen J.
Cusidó, Rosa
Cuypers, Ann
Czaplewski, C.
Czuba, Zenon
D’abrosca, Brigida
D’Angelica, Michael I.
D’argenio, Giuseppe
D’Orazi, Gabriella
D’orazio, John
D’Ovidio, Renato
D’arcy, Pádraig
D’auria, Maurizio
Da Silva, M.L. Alves
Da Silvab, Joaquim C.G. Esteves
Dachs, Gabi U.
Dadlez, Michal
Daeschlein, Georg
Dagna, F.
Dahech, Imen
Dahlberg, Jonas
Dai, Chi-An
Dai, Hongyue
Dai, Qi
Dai, Yao
Daikoku, Takiko
Dall’Olio, Fabio
Dall’Acqua, Stefano
Dalsgaard, Karina
Damia, Giovanna
Dane, Fenny
Dangott, Larry
Daniele, Simoni
Daniele, Stéphane
Danielson, Neil D.
Danilov, Sergei M.
Dansette, Patrick
Darissa, Omar
Das, Saumya
Dasu, Mohan R.
Datta, Geeta
Datta, Tania
Dave, Kunjan R.
David-Cordonnier, Marie-hélène
Davidson, Ben
Davies, Michael
Davies, Terry F.
Davis, Ian
Davis, Randall L.
Davis, Vicki L.
Dawson, Christopher W.
Day, David
Day, Regina
Dayan, Victor
De Alvarenga, E.S.
De Barro, Paul J.
De Berardis, Domenico
De Brevern, Alexandre G.
De Bruijn, Frans J.
De Carvalho, Carla
De Deken, Xavier
De Feo, vincenzo
De Geest, Bart
De Jesús Ornelas-Paz, José
De Jonge, Niels
De Klerk, Arno
De La Fuente, Jesus M.
De La Pompa, José Luis
De La Serna, Ivana L.
De Lourdes Pereira, Maria
De Molina, A. Ramírez
De Ponti, Fabrizio
De Riek, Jan
De Santis, R.
De Vere White, Ralph W.
De Visser, S.P.
Dearden, John C.
Debruyne, Frans
Decho, Alan W.
Dedieu, Stephane
Deevska, Gergana
Defazio, Anna
Deforce, Dieter
Degani, Gad
Degregorio, Michael W
Del Gaudio, Costantino
Delattre, François
Delaurier, April
Den Hertog, Jeroen
Deng, Wuguo
Deng, Yinyue
Deng, Yiqun
Denizot, B.
Dennis, Roger D.
Des Marais, David J.
Desbois, Andrew
Dessaux, Yves
Dessing, Mark C.
Devaney, Eileen
Dewa, Yasuaki
Dhahbi, Joseph M.
Dhar, Animesh
Dhar, Sanjit K.
Dharmananda, Subhuti
Dhillon, Varinderpal
Di Carlo, Marta
Di Girolamo, Nick
Di Maio, Luciano
Di Maria, Emilio
Di Profio, Gianluca
Di Sansebastiano, Gian Pietro
Di Somma, Salvatore
Di, Rong
Diamond, Stephen A.
Dianzani, Umberto
Díaz-Martín, Juan
Díaz-Mínguez, José M.
Diaz, J.
Dick, Gregory M.
Dickinson, M.
Diekwisch, Thomas
Diel, Patrick
Dieplinger, Hans
Dieriks, Birger
Díez-Tejedor, E.
Dilworth, F. Jeffrey
Dimaio Knych, Heather K.
Dimitroff, Charles J.
Dimitroulas, Theodoros
Ding, Wei-Qun
Ding, Wen-Xing
Ding, Yu
Ding, Yuchuan
Dingermann, Theodor
Dini, Luciana
Dinkova-Kostova, Albena
Dinu, Cerasela Zoica
Dinulescu, Daniela M.
Diot, C.
Diretto, Gianfranco
Dirisala, Vijaya
Dirsch, Verena M.
Dittmer, Jurgen
Dixon, Dan A.
Do, Sun Hee
Dobrovolskaia, Marina A.
Dolga, Amalia M.
Dolo, Vincenza
Domenico, Ivana
Domingo-Domenech, Josep
Domingo, C.
Domingos, Pedro M.
Dooley, Steven
Dopp, Elke
Dörffling, Karl
Dorszewska, Jolanta
Dortch-Carnes, Juanita
Dosio, Franco
Douki, Thierry
Doumèche, Bastien
Dowd, Patrick F.
Downes, C. Stephen
Doxsey, Stephen
Drake, A.J.
Dressler, Gregory R.
Drew, Bryan T.
Dreyer, Jean-luc
Drickamer, Kurt
Drissi, Rachid
Drobizhev, Mikhail
Drosten, M.
Drummen, Gregor
Du, Caigan
Du, William Weidong
Du, Xiang
Du, Zhiqiang
Duan, Jicheng
Duan, Jinao
Duarte, T.L.
Dubut, V.
Ducruet, A.F.
Dudás, József
Dudley, Gregory B.
Duennwald, Martin
Duerksen-Hughes, Penelope J.
Duesbery, Nicholas
Duewer, David
Dullaart, Robin P.F.
Dumas, Bernard
Dummer, Reinhard
Duncker, Bernard P.
Dunlevy, Jane R.
Dunsby, Chris
Dupont, Geneviève
Dupré, Denis J.
Durante, William
Duranti, Marcello
Duttaroy, Assim
Dvorak, Zdenek
Dziegiel, Piotr
Dzyuba, Sergei
E. Paul, Cherniack
Eblen, Scott
Eckl, Peter M.
Edelmann, Winfried
Eder, Iris E.
Ederhy, Stephane
Edin, Nina Jeppesen
Edwards, Michael
Edwards, Todd
Efremov, Rouslan
Egloff, Ann Marie
Eguchi, S.
Ehata, Shogo
Ehling, Josef
Eijkelkamp, Niels
Eira, Catarina
Eisenblätter, Michel
Eissner, Günther
Ekblom, R.
El-Alfy, Abir T.
El-Gendy, Nashwa
El-Habr, Elias A.
El-Nezami, Hani
Elazzouzi, H.
Eleftheriadis, Theodoros
Eleni Efstathiou, Eleni
Elhanafi, Ahmad Omar
Ellinger, Dorothea
Ellinger, Jörg
Ellis, Amanda
Elmore, E.
Elsea, Sarah H.
Elviri, Lisa
Emala, Charles W.
Emberton, Mark
Emond, Stephane
Emoto, Noriaki
Enders, Anselm
Endo, Toshiya
Eng, Cathy
Engelberth, Jurgen
Engl, Christoph
Englert, Markus
Enjoji, Munechika
Enrich, Carlos
Eramo, Adriana
Erb, Matthias
Eremin, Sergei A.
Eriksen, Kirsten Thorup
Erné, Ben H.
Erovic, Boban M.
Esashi, Fumiko
Escames, Germaine
Escors, David
Escriva, Hector
Eshoo, Mark W.
Espino, Javier
Espinosa-Urgel, Manuel
Esteve-Romero, Josep
Estévez, Mario
Estrabaud, Emilie
Esvelt, Kevin Michael
Evangelista, L.
Evans, John Spencer
Evans, Joseph L.
Evans, Mark
Evans, Paul
Evron, Tama
Fabbri, Francesco
Fabbro, Alessandra
Fabbro, Massimo Del
Fabek, Sanja
FabriÁs, Gemma
Fagerstedt, Kurt V.
Fahlke, Christoph
Fahmi, Odette
Faik, Ahmed
Fain, John N.
Fairweather, Delisa
Faísca, Patrícia
Fåk, Frida
Falini, Giuseppe
Faller, Roland
Fanali, Gabriella
Fane, Anthony
Fang, Evandro Fei
Fanni, Daniela
Faoro, Franco
Fardilha, Margarida
Faria, Ana
Faria, Tatiane S.
Farooqui, Akhlaq A.
Farquharson, Colin
Farrer, Rhys A.
Faruk, Omar
Fasler, Elizaveta
Fassbender, Amelie
Fath, Melissa
Faul, Christian
Faure, Denis
Favard, Cyril
Favero-Longo, Sergio e.
Favia, Guido
Fechner, Henry
Feinendegen, Ludwig
Feiters, Martin C.
Felding-Habermann, Brunhilde
Fellows, Chris
Felmeyer, Barbara
Felner, Israel
Fenberg, Phillip
Feng, Chia-Hsien
Fenton, Robert
Feo, Francesco
Ferdinandy, Péter
Ferguson, Peter M.
Ferlini, Cristiano
Fernández Laespada, María Esther
Fernández-Briera, Almudena
Fernández-Calotti, Paula
Fernandez-Garcia, Marta
Fernandez-Lafuente, Roberto
Fernández-Mazuecos, Mario
Fernández-Recio, Juan
Fernández-Ruiz, Javier
Fernández-Trujillo, J. Pablo
Fernandez-Zapico, Martin E.
Fernández, Gustavo
Fernández, Mercedes
Feroci, Marta
Feron, Krishna
Ferrándiz-Pulido, C.
Ferrante, Antonio
Ferrari, Stefania
Ferrarini, Alberta
Ferrer, Javier Izquierdo
Ferrer, Montserrat
Ferreras, JosÉ Miguel
Ferri, Nicola
Festekjian, Ara
Fésüs, L.
Fickert, Peter
Field, Erin
Figaszewski, Zbigniew A.
Figg, William D.
Figueiredo, Marxa L.
Figueredo, Carlos Marcelo S.
Filep, Janos G.
Filiou, Michaela D.
Filippi, Jean-Jacques
Filizola, Marta
Fillat, Cristina
Fimia, Gian Maria
Finer, Yoav
Fineschi, Vittorio
Finka, Andrija
Finocchiaro, Gaetano
Fioravanti, Antonella
Fiore, Emilio
Fiorucci, Stefano
Firrao, Giuseppe
Fischer, Marlene
Fischer, Martin C.
Fischer, Tobias W.
Fisher, Urs
Fissell, William
Fita, Ana
Fitches, Elaine C.
Fitó, Montserrat
Fitzgerald, Julia
Fitzkee, Nicholas
Fladung, Matthias
Flamini, Guido
Flanders, Kathleen
Flanders, Kathleen C.
Flechsig, Gerd-Uwe
Fleenor, Bradley S.
Fleisher-Berkovich, Sigal
Flemming, Hans-Curt
Flint, David
Fogolari, Federico
Földes, Gábor
Foletta, Victoria C.
Folk, William
Fonfria, Victor Lorenz
Fong, Yick Wah
Fontana, L.
Foo, Eloise
Fopp-Bayat, D.
Forcada, Jacqueline
Ford, Lance P.
Forli, Stefano
Formisano, Pietro
Fornace, Albert J.
Forrest, J. Craig
Fortunatia, E.
Foss, Brynjar
Foster, J.A.
Foster, Rosemary
Foucher, Fabrice
Fox, Simon
Franceschinis, E.
Francese, Simona
Franchi, Silvia
Francia, Giulio
Franciosi, Elena
Francis, Heather
Franck, Xavier
Franco, Rodrigo
Frangogiannis, Nikolaos G.
Frankel, Daniel
Franková, Lenka
Franzetti, Andrea
Franzke, C.-W.
Frapolli, Roberta
Fredman, Gabriel
Freger, Viatcheslav
Freije, José M.P.
Freitag, Sabine
Frey, Monika
Fridlyanskaya, Irina I.
Friedland, David R.
Friedman, Ran
Fries, Jochen W.U.
Fritsche, Ellen
Froeyen, Matheus
Fröhlich, Eleonore
Fröls, S.S.
Frommhold, David
Frontera, Antonio
Frost, Adam
Fu, Binying
Fu, J.
Fu, Shih-Feng
Fu, Yong-Bi
Fuchs, Michael
Fujii, Takaaki
Fujimori, Akira
Fujimoto, Kiyohide
Fujimoto, Masaru
Fujita, Daisuke
Fujita, Masayuki
Fujita, Tetsuji
Fujiwara, Naoki
Fukada, Yoshitaka
Fukuda, Michiko
Fukuda, N.
Fukui, H.
Fukui, Hiroyuki
Fukumoto, Manabu
Fukushima, Masami
Fulda, Simone
Funahashi, Masahiro
Fung, Kim
Funk, Richard H.W.
Furini, Antonella
Furlong, Fiona
Furness, David N.
Furuichi, Kengo
Furuya, Hideki
Fusco, Vincenzina
Gabai, Vladimir L.
Gailing, Oliver
Gallelli, L.
Gallerani, Raffaele
Galley, H.F.
Gallier, Sophie
Galve-Roperh, Ismael
Gambino, G.
Gameiro, Paula
Gangopadhyay, Nupur N.
Gant, Timothy
Gao, Allen C.
Gao, Grace
Gao, Jing
Gao, Li-Ping
Garab, Győző
Garagna, Silvia
Garbisu, Carlos
Garbutt, Jennie S.
Garcia-Aguilar, Julio
García-Aljaro, Cristina
García-Barrera, Tamara
García-Ortega, Lucía
García-Sánchez, Susana
Garcia, Ana
Garcia, Cyril
Garcia, Rodolfo C.
Gard, Paul
Gardner, Malcolm J.
Garnovskaya, Maria N.
Garovic, Vesna
Garret, Roger
Garten, Antje
Garzón, Javier
Gasparini, Fabio
Gat-Yablonski, Galia
Gatfield, David
Gatto, Barbara
Gaudio, Eugenio
Gava, Alessandra
Gavira, José A.
Gaytan, Francisco
Gazouli, M.
Geballe, Adam P.
Gebay, C.
Gee, Stephen H.
Gehman, John
Gekle, Michael
Gellis, Armand
Genard, Bertrand
Genchev, Georgi
Gentile, Christopher L.
Gentile, P.G.
Gentner, Bernhard
Gentz, Margaret
Georg, Gunda I.
Georgakilas, Alexandros G.
George, Saji
Gerdes, Martin
Gerecke, Kim M.
Germain-Aubrey, Charlotte
German, Michael S.
Gerós, Hernâni
Gescher, Andreas
Geschwind, Jean-francois H.
Gestwicki, Jason E.
Gewirtz, David A.
Ghabrial, Amin
Ghaleh, Bijan
Ghanem, M.E.
Ghanotakis, Demetrios F.
Ghatora, Baljit
Ghavami, Saeid
Ghibelli, Lina
Ghim, Sa-Youl
Ghosh, Jagadananda
Ghosh, Manik
Ghosh, Paramita M.
Ghosh, Santaneel
Giaginis, Constantinos
Giampietro, Philip F.
Giannattasio, Sergio
Giannecchini, Simone
Giannoni, Paulo
Giannotti, Marina I.
Giaouris, Efstathios
Giegé, R.
Gietzen, Dorothy W.
Giguère, Sébastien
Gillies, Elizabeth
Giménez-Gallego, Guillermo
Gimpl, Gerald
Gioeli, Daniel G.
Giordani, Edgardo
Giordano, Piero C.
Giorno, Filomena
Giraudeau, Patrick
Giuliani, Alessandro
Giuliani, Francesco
Glaser, Shannon S.
Gleason, Wallace
Gleeson, John
Glenn, Kevin A.
Gliozzi, Alessandra
Głowacki, Eric Daniel
Gobbi, Alberto
Gobbi, Gabriella
Gobe, Glenda C.
Goberdhan, Deborah C.I.
Goda, Tatsuro
Gohda, Eiichi
Goicoechea, Nieves
Golczak, Marcin
Golding, Michael C.
Goldring, Steven R.
Goldschmidt-Clermont, Pascal J.
Goldsworthy, Michelle
Gollan, Peter J.
Goloverda, Galina
Golz, John F.
Gomes-Pereira, Mário
Gómez, Nazely Diban
Gomez, Nidia
Gondi, Christopher S.
Gonzales-Coloma, Azucena
Gonzalez Muniesa, Pedro
González-Fernández, A.
González-Pacanowska, Dolores
Gonzalez-Polo, Rosa A.
González-Rodríguez, María Victoria
González-Sánchez, M.I.
Gonzalez, Antonio
González, Enrique Abad
González, Miguel
Goodier, John L.
Goodman, A.
Goping, Ing Swie
Gordeladze, Jan Oxholm
Gordon, Sheldon R.
Gorospe, Myriam
Gorshkova, Tatyana
Gorski, Julia J.
Gosselet, Fabien
Goto-Inoue, Naoko
Gotow, Takahiro
Götte, Martin
Gottlieb, Alexandra
Goud, Bruno
Goulão, Luís Filipe Sanches
Gounaris, Antonia
Gousse, Angelo E.
Govindan, Amaranath
Graham, Todd R.
Gram, Lone
Grandori, Rita
Granger, D. Neil
Granica, Sebastian
Granitzer, Petra
Gransee, Heather
Grassi, Gabriele
Grassi, Mario
Grasso, Giuseppe
Grattagliano, Ignazio
Gray, Kelly
Greco, Claudio
Green, Pamela J.
Greenberger, Joel S.
Greenfield, Norma J
Greenwood, Michael T.
Gregg, Brian T.
Gregory, Christopher
Gregory, Richard L.
Greim, Helmut
Greish, Khaled
Greka, Anna
Gridley, Daila S.
Griffith, Wendell P.
Gril, Brunilde
Grimaldi, Maurizio
Grimm, Marcus O.W.
Groll, Andreas H.
Grosa, G.
Grosch, Rita
Gross, Gerhard
Gross, Peter
Groth, Georg
Grötzinger, C.
Grumet, Rebecca
Grumezescu, Alexandru Mihai
Grzelczak, Marek
Grzmil, Michal
Gu, Ji-Dong
Gu, Ruijun
Gualtieri, Maurizio
Guarini, Salvatore
Guedes, Rita C.
Guerau-De-Arellano, Mireia
Guerente, Liliane
Guerra, Davide
Guerrini, Gabriella
Guicciardi, Maria Eugenia
Guil, Sonia
Guillaume, Sophie M.
Guillemin, G.J.
Guillén, J.
Guillot, Thomas
Guillou, Hervé
Gulbins, Erich
Gullbo, Joachim
Gulliver, Linda S.M.
Gulseren, I.
Guo, Junjie
Guo, Zhanhu
Guo, Weixiang
Guo, Xing
Guo, Z. Sheng
Gupta, Sanjay
Gupta, Sanjeev
Gurnett, Christine
Gurzov, Esteban N.
Gutiérrez, S.
Gutterman, David D.
Guzik, Urszula
Guzmán, Carlos
Guzmán, Esther A.
H.E. Hansmann, Ulrich
Haase, Ilka
Habener, Joel F.
Habu, Yoshiki
Hachem, R.Y.
Hada, Megumi
Hadjiargyrou, Michael
Hadjipanayis, Costas G.
Haertlé, Thomas
Hagen, Stephen J.
Hager, Martin D.
Hagerman, Paul
Hahn, Jae
Hahnke, Volker
Haichar, Feth-El-Zahar
Haider, Husnain Kh
Hajirezai, Mohammad-Reza
Hale, Marie
Hall, Edward D.
Hall, Pamela R.
Halloran, Christopher M.
Ham, Jong Hyun
Hamamoto, Ryuji
Hamanishi, Junzo
Hamann, Martine
Hammer, Daniel A.
Hammond, John
Hampp, R.
Han, Amy
Han, Daewoo
Han, J.Y.
Han, Yeon S.
Hanada, Hideki
Hanada, Sanshiro
Handzlik-Orlik, Gabriela
Hannafon, Bethany
Hannun, Yusuf
Hansen, Rikke
Hara, Hiroshi
Hara, Takafumi
Harada, H.
Harazono, A.
Harbinder, Singh
Hardie, Andrew D.
Harding, P.
Hare, Joshua M.
Haring, Robin
Harkness, Troy A.A.
Harmsen, Martin C.
Harries, Lorna W.
Harris, Michael E.
Harris, Randall E
Harris, Randall E.
Harris, Ray
Harris, Tony J.C.
Harrison, Sanjay
Harry, G. Jean
Hart, Joanne
Hartley, John A.
Hartley, Paul S.
Hasanuzzaman, Mirza
Haselberg, Rob
Hashim, Arig Ibrahim
Hashimoto, Kenji
Hashimoto, Takashi
Hashizume, Kouhei
Hashizume, Mineo
Haspel, Nurit
Hassan, Fathi
Hassan, Mohamed
Hassel, Bret
Hatano, Yutaka
Hattori, Noboru
Haus, Erhard L.
Hausmann, Michael
Häussler, Susanne
Hawkridge, Adam M.
Hayashi, Daichi
Hayashi, Takashi
Hayashi, Tomohiro
Haynes, Cole M.
Hazane-Puch, Florence
Hebbard, L.
Hebert, Marc
Hecht, Michael H.
Hecker, Michael
Hecquet, Claudie
Hedoux, A.
Heemers, Hannelore V.
Hefferon, Kathleen Laura
Hegde, Muralidhar L.
Heidari, Mohammad
Heimler, Daniela
Heinäniemi, Merja
Heinlein, Manfred
Heintz, Nicholas
Helgason, Cheryl D.
Hellen, Christopher U.T.
Hellmich, Helen L.
Hendrich, Andrzej B.
Heng, Daniel Y.C.
Hermosín-Gutiérrez, Isidro
Hernandez-Borrell, Jordi
Hernández-Guerra, Manuel
Hernández, J.A.
Hernández, Luis
Hernández, Medardo
Hernandez, Pilar
Herrasti, P.
Herrero-Beaumont, G.
Herrero, Maria-Jesus
Herreros-Villanueva, Marta
Herrmann, Johannes M.
Herzog, Norbert
Heusch, Gerd
Heymans, Stephane
Hickey, Stephen G.
Hidalgo, Juan
Hierro, Aitor
Higashitani, Atsushi
Higgins, Paul J.
Hildebrand, Kevin A.
Hildebrandt, Peter
Hilton, John
Hiltunen, Mikko
Hilvert, Donald
Hintzen, Rogier Q.
Hirahara, Kiyoshi
Hirai, Hirofumi
Hiramoto, Keiichi
Hirano, Katsuya
Hirayama, Takashi
Hiroaki, Fujii
Hirohashi, Yoshihiko
Hirota, Kiichi
Hirota, Shun
Hirotsune, Shinji
Hishida, M
Hitomi, Hirofumi
Ho-Tin-Noé, Benoît
Ho-Yen, C.
Ho, Hong-Nerng
Ho, Hung-Yao
Hodgson, David R.W.
Hoeckner, Martina
Hoene, Miriam
Hoenigl, Martin
Hofacker, Ivo L.
Hofer, A.M.
Hoffman, Robert M.
Hogberg, Helena
Holcik, Martin
Holding, David R.
Holmes, Crystal
Holopainen, Sanna
Holst, Jeff
Holvoet, Paul
Holzinger, Andreas
Honda, Masao
Hondermarck, Hubert
Hong, Chin-Yih
Hong, Fashui
Hong, Jin Tae
Hong, Kyeong-Man
Hong, Yonggeun
Hong, Young-Shick
Honoré, Patrick M.
Honys, David
Hooper, D.C.
Hoozemans, J.J.M.
Hori, Osamu
Hori, Tiago S.
Horie, Masanori
Horn, Simon
Horsley, Laura
Hortelano, Sonsoles
Hoskins, Paul
Hossain, Shakhawat
Hosur, Vishnu
Hou, Weihong
Housecroft, Catherine E.
Howard, O.M. Zack
Howes, Randolph M.
Hoyne, Gerard F.
Hrai, Itaru
Hristoforou, Evangelos
Hsieh, Andrew
Hsieh, Jer-Tsong
Hsing, C.-H.
Hsu, Fu-yin
Hsu, Hsiu-Fu
Hsu, Shih-Hsien
Hu, Guochang
Hu, Kebin
Hu, M.-C.
Huang, Fu-Jen
Huang, Fuzhi
Huang, Gloria
Huang, Hsien-Da
Huang, Jian
Huang, Jiun-Yan
Huang, Jui-wen
Huang, Taosheng
Huang, Wei-Ning
Huang, Yadong
Huang, Yan
Huang, Ying
Huarte, Maite
Hubácek, J.A.
Huber, Thomas
Hudak, Kathrine
Hudzik, Thomas J.
Huh, Jae-Won
Humblot, Vincent
Hummon, A.B.
Hund, Thomas
Hundley, Heather A.
Hunt, Janet
Hur, Dae Young
Hur, Yoonkang
Hura, Tomasz
Hurta, Robert
Husa, Matt
Hussain, Tahir
Hutchinson, Andrew T.
Hüttenhofer, Alexander Georg
Huysmans, Gerard H.M.
Hvidsten, Torgeir R.
Hwa, John
Hwang, Dong-Youn
Hwang, Ki-Chul
Hwang, Thomas I-Sheng
Hyodo, Fuminori
Iacono, E.
Iafisco, Michele
Iba, Michael
Idriss, Hicham
Iglesias, Emilia
Ikari, Akira
Ikeda, Kenichi
Ilott, Nicholas E.
Ilyushina, Natalia A.
Imai, Yasuo
Imaizumi, Akira
Imamura, Fumiaki
Imamura, Masanori
Imaoka, Tatsuhiko
Imesch, P.
Imig, Jochen
Imrie, Corrie T.
Indolfi, Ciro
Innocent, Christophe
Inoue, Hiroyasu
Inoue, Katsutoshi
Inukai, Yoshiaki
Inuzuka, Hiroyuki
Invernizzi, Pietro
Irato, Paola
Irbäck, Anders
Irimura, Tatsuro
Iruela-Arispe, M. Luisa
Isaka, Yoshitaka
Isenberg, Jeff S.
Ishii, Isao
Ishiwata, Toshiyuki
Ishizaki, Takuma
Isidoro, Ciro
Isner, Jean-Charles
Isoda, Katsuhiro
Isokpehi, Raphael D.
Isono, Erika
Istvan, Boldogh
Ito, Hidetaka
Ito, Mayumi
Ito, Seigo
Iusem, Norberto D.
Ivanov, Dimitri A.
Ivanova, Svetlana
Iwamoto, Keisuke S.
Iwamoto, Takashi
Iwasaki, Kenta
Iwasaki, Tomohiro
Izal-Azcárate, Íñigo
Izquierdo, M.
Izumi, H.
Izzo, Lorella
Jaakola, L.
Jaaro-Peled, Hanna
Jackson, Mandy
Jackson, Roy
Jacob, Francis
Jacova, Claudia
Jacquot, Yves
Jafarian-Tehrani, Mehrnaz
Jaffrezic-Renault, Nicole
Jafra, Sylwia
Jain, Abhinav
Jaligot, Estelle
Jambunathan, Kalyani
James, Aaron
Jameson, David
Jamme, Frederic
Janda, Elzbieta
Janda, T.
Jandl, Thomas
Janes, Sam M.
Jang, C.S.
Janitz, Michael
Janowski, Miroslaw
Jansen, Mphm
Jansson, Leif
Jantsch, Michael F.
Jaurand, Marie-Claude
Jean, Ludovic
Jeandet, Philippe
Jeanguenat, André
Jee, Yong-Seok
Jelicks, Linda A.
Jelinek, D.F.
Jelinek, Raz
Jellinger, Kurt A.
Jeng, Yung-ming
Jenkins, Stuart
Jensen, L.H.
Jensen, Lasse
Jenster, Guido
Jeon, Songhee
Jeong, Byeongmoon
Jeong, Seung-Il
Jerónimo, Carmen
Jeschke, Udo
Jeske, Holger
Jeyaseelan, K.
Jha, Mukund
Ji, Lianghui
Jia, Shidong
Jia, Xiao-Bin
Jiang, C.-Y.
Jiang, Shu-Ye
Jiang, Xi
Jiang, Xiaoxu
Jianhui, Qiu
Jimeno, Ciril
Jin, Sha
Jin, Tianru
Jing, Qing
Jing, Xiabin
Jirovetz, Leopold
Jockers, Ralf
Johansson, Christer
Johansson, J.
John P, Alao
Johnson, Casonya M.
Johnson, Erik
Johnson, Greg A.
Johnson, Kristen
Johnson, Xenie
Johnston, Michael
Johnstone, Scott
Joles, Jaap A.
Jones, David N.M.
Jones, Richard Baker
Jorda, Radek
Jordan, Bryen A.
José-Yacamán, Miguel
Joshi-Barve, Swati
Joshi, Harish C.
Joshita, Satoru
Jost, Philipp J.
Joven, Jorge
Joyner-Matos, Joanna
Ju, Yang
Juan, Hsueh-Fen
Juillerat-jeanneret, Lucienne
Jullien, F.
Jung, Christof
Jung, Hyungtaek
Jung, Klaus
Jung, Marianna E.
Jung, Seunho
Jung, Youngmi
Jurat-Fuentes, Juan Luis
Juszczak, M.
Jutooru, Indira
Jutras, Brandon L.
Juttner, Friedrich
Juzeniene, Asta
K. Deyholos, Michael
Kabelitz, Dieter
Kadowaki, Norimitsu
Kaguni, Jon M.
Kai, Keita
Kaji, Hirokazu
Kajitani, Takashi
Kaklamani, Virginia
Kalies, Kai-Uwe
Kalmar, Bernadett
Kalonia, Devendra
Kalthoff, Holger
Kaludercic, Nina
Kamiya, Hidehiro
Kamiya, Shigeru
Kamolz, Lars
Kan, Lixin
Kanaoka, Masahiro
Kanauchi, Osamu
Kandouz, Mustapha
Kandror, Konstantin V.
Kandzari, S.J.
Kang, Byung-Ho
Kang, Chi-Dug
Kang, Chulhun
Kang, Hee Eun
Kang, Jihong
Kang, Li
Kang, Min H.
Kangueane, Pandjassarame
Kannanagattu, Prasanth
Kantharidis, Phillip
Kapiloff, Michael S.
Kapoor, Mohit
Kappen, Claudia
Kapus, András
Karabencheva, Tatyana
Karagiannis, Tom C.
Karatan, Ece
Karavitakis, Markos
Karjalainen, Reijo O.
Karlovsky, Petr
Karlsson, Jan Olof G.
Kasarda, Don
Kashiwagi, Satoshi
Kashyap, Rahul
Kasper, Siegfried
Kästner, Johannes
Katayama, Tsutomu
Kato, Hirohito
Kato, Kikuya
Kato, Mikio
Kato, Tomohiro
Katsaras, John
Katsuta, Shoichi
Katus, Hugo
Kaufman, Laura
Kaul, Marcus
Kaur, Sarabjot
Kauss, Tina
Kawada, Manabu
Kawai, F.
Kawamori, Toshihiko
Kawamura, Kazuhiro
Kawasaki, Hideki
Kaymak, Haluk Çağlar
Kazakov, Andrey
Kazarian, S.
Kazi, Abid
Kazuhito, Satomura
Ke, Ya
Keck, Bastian
Keep, Richard
Keim, N.L.
Kelley, Darshan S.
Kelly, Joan M.
Kemeny, Nancy E.
Kemp, Kevin
Kemper, Byron
Kenmochi, Naoya
Keogh, Brian
Kerkeni, Mohsen
Kerkhoff, Claus
Kerr, Thomas
Kersey, Paul J.
Kersten, Sander
Kesavalu, L.
Kessel, David
Keszenman, Deborah
Keul, Helmut
Kevil, Christopher
Kevill, Dennis
Keyser, Ulrich F.
Khaliulin, Igor
Khan, Amir R.
Khan, Anzar
Khan, Muhammad
Khanna, Savita
Khare, Sharad
Khemtémourian, Lucie
Khraiwesh, Basel
Khurana, Sandeep
Kiddle, Guy
Kidoaki, Satoru
Kiemer, Alexandra
Kiesslich, Tobias
Kietzmann, Thomas
Kikuzaki, Hiroe
Kiltie, Anne
Kim, Bhumsoo
Kim, Byung-Hoon
Kim, Cheorl-Ho
Kim, Chul Young
Kim, Eui-Chong
Kim, Han Sung
Kim, Hee Sung
Kim, Heon
Kim, Hugh I.
Kim, Hyon-Suk
Kim, In Kyeom
Kim, Jae Bum
Kim, Jae-Young
Kim, Jayoung
Kim, Jin Kyeoung
Kim, Jin-Soo
Kim, Jong Hwa
Kim, Junhwan
Kim, Kyuseok
Kim, Mee Ree
Kim, Minho
Kim, Moon Suk
Kim, Sang-We
Kim, Sun
Kim, Sung Won
Kim, Sung-Hoon
Kim, Tae-wuk
Kim, Uk-Kyu
Kim, Wun-Jae
Kimata, Yukio
Kimitsuki, Takashi
Kimmig, Rainer
Kimple, Randall J.
Kimura, Hideo
Kimura, Ikuo
Kindt, James T.
Kindy, M.S.
King, Charles C.
Kinoshita, Manabu
Kirimura, Kohtaro
Kirker, Kelly
Kirkham, Matthew
Kirschstein, Timo
Kishan, Amar U.
Kiss, Anna K.
Kiss, Eva
Kitagawa, Fumihiko
Kitajima, Shigetaka
Kjellsen, Trygve Devold
Klausen, Christian
Kleczkowski, Leszek A.
Kleeff, Jörg
Klein-Nulend, Jenneke
Klein, Eva
Klein, Ronald L.
Kleinman, Hynda K.
Kleszczyński, Konrad
Klink, Vincent P.
Klitgaard, Kirstine
Klokov, Dmitry Y.
Klopfleisch, R.
Klopfleisch, Robert
Klosterman, Steven J.
Kluza, J.
Kneitz, S.
Kneteman, Norman
Knipper, Marlies
Knizetova, Petra
Knoop, Volker
Knopp, Dietmar
Ko, Jiunn-Liang
Kobayashi, H.
Kobayashi, Motoyasu
Kobayashi, Yukiho
Kobeissy, Firas H.
Kobori, Hiroyuki
Koch, Tad H.
Kocsy, Gabor
Kodavanti, Urmila
Koeckerling, Martin
Kofuji, Kyouko
Koh, David W.
Kohli, Ajay
Koide, Hikaru
Kojima, Chie
Kojima, Shihoko
Kojima, Takashi
Kojo, Shosuke
Kok, C.
Kolesnichenko, Vladimir L.
Kolialexi, Aggeliki
Kolo, Kamal
Koltai, Hinanit
Komers, Radko
Komis, George
Kondyurin, Alexey
König, Janine
König, Jörg
Konigsberg, William H
Konradsen, Jon R.
Konstantinopoulos, Panagiotis A.
Kooyman, David L.
Kopra, Kari
Korbel, Jan O.
Koriyama, Chihaya
Korol, Donna
Korsching, Eberhard
Kosmala, Arek
Kostin, Sawa
Kota, Satya K.
Kothe, Erika
Koti, Madhuri
Kovalainen, Miia
Kovinich, Nik
Koya, Daisuke
Koynov, Kaloian
Kozak, Maciej
Kozono, David
Kozytska, Svitlana
Kracht, Michael
Kramer, Koen
Kraus, Robert H.S.
Krause, Kirsten
Krenács, Tibor
Kressler, Jörg
Krueger, A. Chris
Krüger, Rejko
Kruszelnicka, Olga
Kubicek, Christian P.
Kubis, Nathalie
Kubo, T.
Kubo, Tomohiko
Kubota, Satoshi
Kudera, Stefan
Kuehn, Christian
Kuhla, Angela
Kuhlemeier, Cris
Kuktaite, Ramune
Kukuruzinska, Maria A.
Kuliawat, Regina
Kulkarni, Chandrashekhar
Kulkarni, Chandrashekhar V.
Kulkarni, Rahul
Kumagai, Kousuke
Kumamaru, Toshihiro
Kumar, Ajit
Kumar, Sanjai
Kumar, Santosh
Kumazawa, Yoshinori
Kumbhar, Anupa
Kumita, Janet
Kunimasa, Kazuhiro
Kunos, Charles
Kunze, Doreen
Kunze, R.
Kuo, Cheng-Deng
Kuramitsu, Seiki
Kurihara, Hideyuki
Kurihara, Tatsuo
Kurokawa, Riki
Kurose, Hitoshi
Kurre, Peter
Kushibiki, Toshihiro
Kuwasako, Kenji
Kuwata, Keith T.
Kuželová, Kateřina
Kveberg, Lise
Kvist, S.
Kwak, Jong-Young
Kwon, Guim
Kwon, Hawk-Bin
Kwon, Youngjoo
Kwong, Joseph
Kyong, Jin Burm
Kyprianou, Natasha
Kyzas, George
La Bella, Vincenzo
La Mesa, Camillo
La, Young-Hye
Laaksonen, M.A.
Labate, Joanne A.
Labbe, Simon
Lacomme, Christophe
Ladd, Andrea N.
Lai, Chunze
Lam, Hon Ming
Lam, Kit S.
Lam, Koon Fung
Lamartine, Jérôme
Lambert, Elisabeth
Lampert, Angelika
Lan, Zhiyuan
Lanaspa, Miguel A.
Landen, Charles N.
Landis, J.B.
Landreville, Solange
Landry, Veronic
Langdon, Simon P.
Langenberg, D.R. Van
Langsley, Gordon
Lapres, John J.
Lara, Isabel
Laronde-Leblanc, Nicole
Laronga, Christine
Larrainzar, Estibaliz
Larsen, Anders Peter
Larson, Benjamin
Lartigue, Lenaic
Larue, Valéry
Laschke, M.W.
Lascialfari, Alessandro
Lash, G.E.
Lassau, Nathalie
Lassegue, Bernard
Latef, Aaha
Latella, Giovanni
Latour, Xavier
Latrofa, Francesco
Lattuada, Marco
Lau, K.H. Aaron
Lau, Steffen
Laukkanen, Mikko O.
Laurencin, Cato T.
Lausen, Jörn
Lavrik, Inna N.
Lazzara, Giuseppe
Le, Dzung Tien
Le, Yuan
Leadbeater, Nicholas E.
Leak, Rehana K.
Leask, Andrew
Lebaron, Richard
Lee, Beth S.
Lee, Dong-ki
Lee, Dongjin
Lee, Eunjoo H.
Lee, Gabsang
Lee, Hyun Soon
Lee, Inhan
Lee, J.-H.
Lee, Jin-Yul
Lee, Jongmin
Lee, Ju-Seog
Lee, Lance
Lee, Mi Kyeong
Lee, Ming-Fen
Lee, Ming-Shyue
Lee, Natuschka M.
Lee, Ok-Hwan
Lee, Rita S.F.
Lee, Sae-Won
Lee, Sang Kook
Lee, So Yeong
Lee, Taeseung
Lee, Tzong-Shyuan
Lee, Wenjau
Lee, Wooin
Lee, Y.S.
Lee, Yong Sun
Lee, Yun-Shien
Lees, Watoson J.
Leffak, Michael
Legler, Daniel F.
Legname, Giuseppe
Lehtonen, Sanna
Leidinger, Petra
Leisner, J.J.
Leitch, I.J.
Lemke, Edward A.
Lemonnier, Loic
Lenaz, Giorgio
Leng, Shuguang
Lenoir, G.
Lentz, Steven
Leo, Oberdan
Leong-Poi, Howard
Leoni, Livia
Leosco, Dario
Lepenies, Bernd
Lepore, Maria
Lescaudron, Laurent
Lessard, Christopher
Leu, Hsin-Bang
Leubner-Metzger, Gerhard
Leuner, Kristina
Leung, Joseph C.K.
Levin, Robert E.
Levkau, Bodo
Levon, Kalle
Lewy, Alfred J.
Leys, David
Lhiaubet, Virginie Lyria
Li Volti, Giovanni
Li, Albert P.
Li, Ang
Li, Bing
Li, Chengdao
Li, Edmond C.K.
Li, Fan
Li, Genxi
Li, George Qian
Li, Huige
Li, James C.B.
Li, Jing
Li, Jingjing
Li, Li
Li, Mao
Li, Pei-Feng
Li, Qing Kay
Li, Ren-Ke
Li, Robert W.
Li, Rugang
Li, Tiangang
Li, Yefu
Li, Yi
Li, Yinxin
Li, Yong
Li, Zhigang
Liang, Carol Jiurong
Liang, Chia-Hua
Liang, Po-Huang
Liang, Willmann
Liang, Xue-Hai
Liao, Chien-sen
Liao, Pao-Chi
Liao, Shuen-Kuei
Liapi, Charis
Lien, Jin-Cherng
Liesegang, A.
Lillehaug, Johan R.
Liloglou, Triantafillos
Lim, Yong-Beom
Lima, Emerson Silva
Limoli, Charles L.
Lin, Chi-Chen
Lin, Ching-Shwun
Lin, Ching-Yu
Lin, Chiou-Feng
Lin, Chun-Rong
Lin, Haocheng
Lin, Hsu Y.
Lin, Hung-Yin
Lin, Hungdu
Lin, Jiang-Jen
Lin, Jiann-T’suen
Lin, Jin-Yuarn
Lin, Nianwei
Lin, Shiu-Ru
Lin, Tao
Lin, Yiwei
Lin, Yun-Lian
Lin, Zu-Yau
Lindberg, Raija
Linde, G.A.
Lindner, Uri
Lindner, Volkhard
Lindstrom, Anders J.
Linert, Wolfgang
Ling, Yong-Chien
Linkermann, Andreas
Lipkowski, Jacek
Lipton, Allan
Litosch, Irene
Little, R. Daniel
Littleton, R.M.
Liu, Alvin Y.
Liu, Brian C.-S.
Liu, Chong
Liu, Chunyu
Liu, Delong
Liu, Dongmin
Liu, Fen-Yong
Liu, Feng
Liu, Guei-Sheung
Liu, Hongyu
Liu, Jah-Yao
Liu, Jiang-Xun
Liu, Jiaren
Liu, Juewen
Liu, Jun-Qi
Liu, Junqiu
Liu, Shing Hwa
Liu, Shufang
Liu, Shuliang
Liu, Tao
Liu, Xuelai
Liu, Yang
Liu, Yi-Wen
Liu, Yung-Chuan
Liu, Zhao-Jun
Liu, Zhenguo
Lladó, Jerònia
Lo Brutto, S.
Lo, Amy C.Y.
Lo, S.S.
Lobo-Castañón, Maria Jesus
Loeffler, Hannes H.
Loeser, R.F.
Loessner, Daniela
Loewen, Michele C.
Loewen, Peter C.
Lohr, Jamie L.
Lojou, E.
Lokshin, Anna
Lokshin, Anna E.
Lombardo, G.
Lomoth, Reiner
Lopato, Sergiy
Lopes, Maria Ascensão
Lopes, Maria De Fátima Silva
Lopez-Beltran, Antonio
López-Gómez, Miguel
Lopez-Lluch, Guillermo
López-Ráez, Juan
Lopez, Secundino
Loppi, S.
Lorenz, Kristina
Lorenzo, Oscar
Lorite, I.
Losano, Gianni
Lou, Lili
Louhi, Katja-Riikka
Lowe, Martin
Loyer, Pascal
Lpez-Legentil, Susanna
Lu, Kuo-Cheng
Lu, Ming-Wei
Lu, Wejie Jessie
Lübeck, Mette
Lucarelli, Giuseppe
Lucau-Danila, A.
Luciani, Nathalie
Luckarift, Heather R.
Ludwiczuk, Agnieszka
Ludwig-Müller, J.
Ludwig, Frank
Lui, Vincent Chi-Hang
Luk, Yan-Yeung
Lund, Riikka
Lundén, Karl
Lunov, Oleg
Luo, Jian-Hua
Luo, Ming-cheng
Luo, Zhenghua
Lupiáñez, José A.
Lutfy, Kabirullah
Lutz, Pierre G.
Luzza, Francesco
Lymperopoulos, Anastasios
Lynch, Cian J.
Łysakowska, Monika Eliza
Lyubchenko, Yuri L.
Ma, Chao-Mei
Ma, Chenming
Ma, Edmond Dik-Lung
Ma, Fengwang
Ma, Hongming
Ma, Jinfang
Ma, Liyuan
Ma, Shisong
Ma, Yu-Qiang
Maas, Amy E.
Macchi, Paolo
MacDonald, Ruth
Machaalani, Rita
Machamer, Carolyn
Machelon, Véronique
Maciel, Patrícia
Madej, Andrzej
Madeline, Torres-Lugo
Madhavan, Dharanija
Madigan, Michele
Maeda, Shiro
Maffei, F.
Magali, Terme-Zydel
Magne, D.
Maher, Pamela
Mahimainathan, Lenin
Mahmoud, Soheil S.
Maïbèche-Coisne, Martine
Mailloux, Ryan J.
Maisch, Tim
Majumdar, Adhip
Makarov, Denys
Mäkinen, K.K.
Makkonen, Jenny
Malaguarnera, Mariano
Maldonado, Maria M.
Malemud, Charles J.
Malfatti, Michael A.
Malik, Afshan N.
Malik, Vikash
Malik, Zvi
Mamedov, Tarlan
Mamiya, H.
Mamone, Gianfranco
Mandal, Subhrangsu S.
Mandel, Karl
Mandic, Milica
Manfredi, Kirk P.
Manga, Prashiela
Mangge, Harald
Mangoni, Arduino
Mani, Sridhar
Mannell, Hanna
Manolopoulos, Vangelis E.
Mantell, C.
Manuel, Michele V.
Manzano-Román, Raúl
Mao, Chengde
Mapelli, Marina
Maquat, Lynne E.
Marchesan, Silvia
Marco-Contelles, José
Marcovici, G.
Marcucci, Fabrizio
Marfaritis, Marios
Margaritis, Lukas H.
Maricle, Brian R.
Marie, Pierre J.
Marimpietri, Danilo
Marín, Francisco
Marin, Jose J.G.
Marini, Frank C.
Marini, Juan C.
Marion, Sandrine
Mark, Alan E.
Markowitz, Joseph
Marks, David
Markwardt, Fritz
Marmiroli, Nelson
Marmorstein, Alan D.
Maroja, Luana S.
Marquis, Christopher
Marsh, Deborah J.
Marszałł, Michał Piotr
Martens-Uzunova, Elena S.
Martín-Aragón, Sagrario
Martinelli, Adriano
Martínez-Chantar, Maria L.
Martínez-Cuesta, M. Carmen
Martínez-Felipe, Alfonso
Martínez-Fernández, Mónica
Martinez-Fong, Daniel
Martínez-Gómez, Pedro
Martínez-Haya, Bruno
Martínez, Alfredo
Martinez, Inigo
Martinez, Javier
Martinez, Karen L.
Martinez, Manuel
Martino, Sabata
Martins, L. Miguel
Maruyama, Tetsuo
Marvar, P.J.
Mas, Sebastián
Mason, Hugh
Mastaloudis, Angela
Mastbergen, S.C.
Mastrangelo, Anna M.
Masuda, Yuki
Masumori, N.
Masumoto, Junya
Masuo, Kazuko
Masutomi, Kenkichi
Mateos, Carlos
Mateos, Pedro
Matés, José M.
Mathesius, Ulrike
Matos, Ana Rita
Matsagkas, M.I.
Matsumiya, Masahiko
Matsunaga, Sachihiro
Matsunaga, Tadashi
Matsuno, Koji
Matsuo, Takashi
Matsuoka, Daisuke
Matsuoka, Masaaki
Matsuoka, Masato
Matsushita, Yumi
Matsuura, Kazunori
Matteucci, Andrea
Matuszewski, Łukasz
Matzuk, Martin
Maughan, P.J.
Maurino, Veronica Graciela
Mauriz, José L.
Mauro, A.M. Carai
Mavelli, Irene
Max, Nelson
Mayama, Hiroyuki
Maycock, Christopher D.
Mayer, Jens
Mayer, Klaus
Mayr, Johannes A.
Mayr, Manuel
Mazza, Monica
Mcanulty, Steve
Mcbain, Andrew
Mcconnell, Michelle
Mccormick, Peter J.
McDaneld, T.G.
McDermott, Suzanne M.
Mcewan, Iain
McFeeters, Robert L.
McGill, Mitch
Mcgrath, Sophie E.
Mclntosh, Michael
Mcloon, Linda K.
McMartin, Kenneth
McMeekin, D. Scott
Mcnealy, Tamara
Meade, Thomas J.
Mechref, Yehia
Medeiros, Rui
Medina-Gomez, Gema
Meimberg, H.
Meinlschmidt, G.
Meins, Jean-François Le
Meldolesi, Jacopo
Meliska, Charles J.
Melke, Jonas
Mellick, Albert S.
Meloni, Bruno P.
Mendes, N.D.
Méndez-Sánchez, Nahum
Meneghetti, F
Menéndez, Alejandro Cuetos
Meng, Kun
Meng, Xiaojie
Meng, Xue Wei
Mengoni, A.
Menna, Elisabetta
Menschikowski, Mario
Menten, B.
Merchant, Naaz
Merfort, I.
Meshorer, Eran
Meyer, Christian
Meyer, Giuliano
Meyer, Michelle
Meyer, Ralph
Mezey, Eva
Mezzanzanica, Delia
Mhawech-Fauceglia, Paulette
Miaskowski, Christine
Miccadei, Stefania
Michael, Agnieszka
Michaeu, Olivier
Michel, Andrew P.
Michel, R.N.
Micheli, D.
Michelozzi, Marco
Mico, Juan A.
Midde, K.
Miedaner, Thomas
Mielczarek, C.
Mielenz, M.
Migliore, Lucia
Mignet, Nathalie
Miguel, M.G.
Mileykovskaya, Eugenia
Miller, Dylan V.
Miller, Grant G.
Miller, Jason R.
Miller, Leland V.
Miller, Stephen T.
Milne, Kevin
Milner, Peter I.
Minami, Yoshiyasu
Minamoto, Toshinari
Minden, Audrey
Minetti, Giampolo
Ming, Mei
Minguez, guillermo
Minoia, Carla
Minoo, P.
Minucci, Sergio
Miosge, Nicolai
Miquel, M.
Mir, Mustafa
Miranda, Adrian
Misra, Devesh
Mita, Damiano Gustavo
Mitani, Akio
Mitchell, Brett M.
Mitchell, Tom
Mitchison, Timothy J.
Mithöfer, Axel
Miura, K.
Miura, Osamu
Miyamoto, Hiroshi
Miyamoto, Takeshi
Miyao, Toshihiro
Miyata, Yasuyoshi
Miyoshi, Norio
Mizoi, Junya
Mizuguchi, Junichiro
Mizuo, K.
Mlotshwa, Sizolwenkosi
Moberg, Kenneth H.
Mocellin, Simone
Moeslinger, Thomas
Mognato, Maddalena
Moguel, Mcm
Mohamadzadeh, Mansour
Mohamedali, Khalid
Mohler, James L.
Mohn, Angelika
Moioli, Bianca
Moiseyev, Gennadiy
Mok, Ka Pun Chris
Mokili, John L.
Molasiotis, Athanasios
Molday, Robert S.
Moldovan, George-Lucian
Molin, Daniel G.M.
Moliner, Vicent
Molle, Virginie
Möller, Harald
Molnár-Láng, Márta
Molnar, Arpad
Molvarec, Attila
Monge, Miguel
Mongillo, Marco
Monleon, Daniel
Monte, Enrique
Monzon, Lorena M.A.
Moon, Yuseok
Moore, Richard G.
Moore, Tara C.B.
Morais-Cecílio, Leonor
Morales, M.P.
Moran, Nancy
Moran, Yehu
Moreira, Eduardo L.G.
Moreno-Herrero, Fernando
Moreno, María U.
Morgan, Reginald
Morgan, Richard
Morgillo, Floriana
Mori, Hirotoshi
Mori, Naoki
Moriconi, Federico
Morillon, R.
Morioka, Norimitsu
Morishita, Yoshiyuki
Morisseau, Christophe
Morita, Kyoji
Morita, Shigeto
Moriwaki, Hisataka
Morley, Alexander A
Moro, L.
Morohashi, Ken-Ichirou
Morohoshi, Tomohiro
Möröy, Tarik
Morris, Brian J.
Morris, David R.
Morrison, Janna L.
Morrison, Wayne A.
Moscardó, Antonio
Moschou, Panagiotis N.
Moser, Jürgen
Mosesso, Pasquale
Mostafavi, Mehran
Motherway, Mary O’connell
Mott, Bryan T.
Motta, Antonella
Motterlini, Roberto
Motti, Cherie
Mouliere, Florent
Mounsey, Kate E.
Mouraviev, Vladimir
Mourtada-Maarabouni, Mirna
Mousa, Shaaban A.
Moussa, Omar
Mu, Dezhi
Mu, Yuguang
Mudryj, Maria
Mueller-Roeber, Bernd
Mühlenhoff, Ulrich
Muise-Helmericks, Robin C.
Mukherji, Mridul
Mukhopadhyay, Rita
Mukohara, Toru
Mulè, F.
Muleo, Rosario
Mulo, Paula
Muneer, Asif
Munekazu, Yamakuchi
Muñoz-Barroso, Isabel
Muñoz-Bonilla, Alexandra
Muñoz, Luis
Münsterberg, Andrea
Murai, Koji
Murakami, Yasufumi
Murakami, Yoshiki
Murashita, Koji
Murata, Jin
Murata, Kenji
Murata, Norio
Murata, Yoshiyuki
Muratore, Andrea
Mürdter, Thomas E.
Murga, C.
Muro, Manuel
Murphy, Andrew
Murphy, Madeline
Murray, Graeme I.
Murray, Lynne A.
Murugaiyan, Jayaseelan
Musco, Giovanna
Musilek, Kamil
Musso, O.
Mustjoki, Satu
Musumeci, Teresa
Musunuru, Kiran
Muyldermans, Serge
Muzio, Giuliana
Myöhänen, Timo T.
Myung, Pyung-Keun
N. Frick, David
Nadal-Desbarats, Lydie
Nadiminty, Nagalakshmi
Naftalin, Richard
Nagahara, Yukitoshi
Nagamitsu, Tohru
Nagao, S.
Nagarajan, Shanmugam
Nagata, Eiichiro
Nagata, Shigekazu
Nagatoishi, Satoru
Nagel, Stefan
Nagy, Milan
Nair, Ashwin
Nair, Satish K.
Naito, Kiyohito
Nakagomi, Takayuki
Nakahama, Kenichi
Nakahara, Kenji S.
Nakajima, Miki
Nakaki, Toshio
Nakamichi, Norihito
Nakamura, Hiroaki
Nakamura, Kazufumi
Nakamura, Nobuhiro
Nakamura, Soichiro
Nakamura, Takehiro
Nakano, Atsushi
Nakashima, Kazuo
Nakata, Satoshi
Nakatomi, Akiko
Nakayama, Kohzo
Nakazawa, Futoshi
Nalam, Vamsi
Nalivaeva, Natalia
Nam, Kyoung Hee
Namba, Shigetou
Namgaladze, Dmitry
Namura, Shobu
Nangmenyi, Gordon
Nanjundaiah, S.
Nanjundan, M.
Nansen, Christian
Naoi, Makoto
Napierala, Marek
Narain, Ravin
Narayan, Mahesh
Nardelli, Carmela
Naruse, Keiji
Nastruzzi, Claudio
Nasu, Kaei
Nater, Alexander
Natile, Giovanni
Natsugoe, Shoji
Navara, Christopher
Navarra, Michele
Naviglio, Silvio
Nawa, Katsuhiko
Naya, Francisco J.
Nazzaro, Filomena
Negrão, Sónia
Negri, Rodolfo
Neimark, Alexander V.
Neira, José L.
Neirinckx, Virginie
Nejak-Bowen, Kari N.
Nekhai, Sergei
Nemoto, Edwin M.
Nemunaitis, John
Nerurkar, Pratibha V.
Nervi, Clara
Neureiter, Daniel
Neves, Ricardo
Newman, Robert A.
Ng, Tzi
Ng, Yin Kwee
Nguyen, Phuong
Nguyen, Tuan.
Nicholson, Wayne L.
Nicolas-Molina, F.E.
Niculescu, Violeta-Carolina
Niessen, carien
Nieto, Laurence
Nieves, Edward
Nigris, D.E.
Niibe, Yuzuru
Niidome, Takuro
Nikinmaa, Mikko
Nikolic, Dragana B.
Nilsen-Hamilton, Marit
Nilsson, Å.
Nilsson, Jan
Nilsson, Staffan
Ninov, Nikolay
Nischwitz, S.
Nishio, Z.
Nishizaki, Tomoyuki
Nishizawa, Kazuhisa
Nisman, Benjamin
Nissen-Meyer, Jon
Nitschke, W.
Nobile, Mario
Noboru Katayama, Noboru
Noessner, Elfriede
Nofer, Jerzy-Roch
Noguchi, Constance Tom
Nogué, F.
Nolta, Jan A.
Nonn, L.
Nordberg, Gunnar
Norman, Trevor R.
Normand, Sylvain
Norris, Andrew W.
Nostro, Maria Cristina
Novella, Susana
Novo-Uzal, Esther
Nowak, Julia S.
Nowicki, Michal
Nozik-Grayck, Eva
Numakawa, Tadahiro
Nusayr, Eyad
Nussler, Andreas
Nwariaku, F.E.
Nyanhongo, Gibson S.
Nylander, Karin
O’Brien, Katie M.
O’Keefe, Stephen JD
O’connell, Mary A.
Obana, A
Obe, Guenter
Obermeier, C.
Obrenovich, Mark E.
Ochi, Mitsukazu
Ochiai, M.
Ochiya, Takahiro
Oehme, Ina
Oger, Phil M.
Ogino, Shuji
Ogita, Kiyokazu
Ogle, Brenda M.
Oh, Chang-Sik
Oh, Dong-Ha
Oh, Eok-Soo
Ohe, Kenji
Ohlendieck, Kay
Ohnishi, Kouhei
Ohta, Kunimasa
Ohta, Shigeo
Ohta, Yoshiji
Ohuchida, Kenoki
Okada, Masahiro
Okamoto, Takashi
Okamura, Noboru
Okazaki, Toshiro
Okazawa, Atsushi
Okello, E.J.
Okuno, Tsuyoshi
Olcese, James
Olive, M. Foster
Oliver, Mel
Olmos, Enrique
Olsen, N.J.
Olson, W.K.
Olza, Josune
Onichtchouk, Daria
Ono, Koh
Ono, Motoharu
Ooms, Karen
Oorni, Katariina
Oostenbrink, Chris
Oosterwijk, Egbert
Opstad, Kirsti
Ordog, Tamas
Orita, Yorihisa
Orlandi, Augusto
Orozco, Jahir
Ortiz, Inmaculada
Osaka, Tetsuya
Osawa, Tsuyoshi
Osman, Tarig A.
Ospina Millán, Claudia Andrea Ospina
Ostenfeld, Marie Stampe
Ostersetzer-Biran, Oren
Otting, Gottfried
Ouchi, Yasuyoshi
Oukarroum, Abdallah
Oulahal, N.
Oulahal, Nadia
Overmoyer, Beth
Ozaki, Iwata
Özkaya, Ali Rıza
Paclet, M.H.
Padillo, Franscisco J.
Pados, G.
Paduch, Roman
Pagano, Mario Angelo
Pagnan, Gabriella
Pagnotta, Augusto
Pain, Bertrand
Pajonk, Frank
Pakdel, Farzad
Palacios, José
Pallauf, Kathrin
Palm, Gottfried J.
Palmieri, Beniamino
Palstra, S.W.L.
Palza, Humberto
Pampalakis, Georgios
Pan, Hung-Chuan
Pan, Min-Hsiun
Pan, Tai-Long
Panchatcharam, Manikandan
Pandey, Amit V.
Pandey, Manoj
Pandey, Udai Bhan
Pandolfi, Paolo
Panfoli, Isabella
Pang, Lisa
Pang, Yuan-Ping
Panitz, Frank
Paniwnyk, L.
Pantopoulos, Kostas
Paolocci, Francesco
Paolocci, Nazareno
Papaccio, Gianpaolo
Papachristou, D.J.
Papadimitriuou, Konstantinos
Papadopoulou, Kalliope K.
Papageorgiou, Sotirios
Papageorgis, Panos
Papaiconomou, Nicolas
Papaleo, Elena
Papp, Tamás
Pappa, Aglaia
Pariente, J.A.
Parisi, Silvia
Park, Cheon-Seok
Park, Il-Kwon
Park, Ji Sook
Park, Jong
Park, Jong-Chul
Park, Jongsun
Park, Joo-Cheol
Park, Junsoo
Park, Ky Young
Park, Phun Bum
Park, SungWoo
Park, Raekil
Park, S.H.
Park, Sung Young
Park, Sungha
Park, Woo-Yoon
Park, Woong June
Park, Yong-Koo
Parker, J. Alex
Parker, Jane K.
Parl, Fritz F.
Parmar, Sachin
Parola, Maurizio
Parsley Walker, Larissa
Parthasarathy, Sampath
Pasc, Andreea
Paschalidis, Konstantinos
Pasinetti, Giulio M.
Passarino, Giuseppe
Pastor, Victoria
Patankar, Manish S.
Patching, Simon G.
Patel, Avignat
Patterson, Darrell A.
Patterson, Karen C.
Pattison, David
Paul, Matthew
Paulmurugan, Ramasamy
Pautz, Andrea
Pavez Lorie, Elizabeth
Pavone, F.S.
Pawar, Snehalata
Pawlak, Dariusz
Pawlowski, Katharina
Payne, Christina M.
Pazzagli, L.
Pecina-Quintero, Víctor
Pedrazzini, Thierry
Peinado, Héctor
Pelekanou, Vassiliki
Pellegrini-Giampietro, Domenico E.
Pena, Romi N.
Penalva, Luiz O.F.
Pendu, Jacques Le
Peng, Hanjng
Peng, Ji-Bin
Peng, Robert Y.
Peng, Yu-Sen
Penn, Raymond B.
Penning, Louis
Pennypacker, Keith
Peppelenbosch, Maikel P.
Peralta, D.R.
Perán, Macarena
Pereira, M. Alcina
Perera, Ranjan J.
Perez, Franck
Perez, Miguel Lopez
Perreau, Victoria M.
Perretti, Mauro
Perrone, Jens
Perry, Antoinette
Perry, Mark
Persad, Raj
Persson, Jenny L.
Perthame, Benoit
Perticone, Francesco
Perucka, Irena
Peschel, Andreas
Peschke, Elmar
Peters, Ulf
Peterson, Daniel
Peterson, Daniel G.
Peterson, Pärt
Pethrick, Richard A.
Petrache, Horia I.
Petrey, Donald
Petric, Ines
Petrova, Olga
Pettersson, Sven
Petzelbauer, Peter
Peuhkuri, Katri
Pfaffl, Michael W.
Pfeifer, Gerd
Pfister, Sandra L.
Philip, Benjamin N.
Philip, Nisha
Phillips, Chris C.
Phillips, Joanna
Piazza, Gary
Picciani, Paulo H.S.
Pichová, Iva
Picken, Maria M.
Pickles, Raymond J.
Picklo, Matthew J.
Piepho, Hans-peter
Pierce, Grant N.
Pignata, Sandro
Pijut, Paula M.
Pikus, Stanislaw
Pilbrow, Anna P.
Pimentel, Gustavo D.
Pin, Lim
Pinazo-Durán, Maria D
Pineda, M.
Pinheiro, Carla
Pinteaux, Emmanuel
Pinzani, Massimo
Pio, Ruben
Pisetsky, David S.
Pissuwan, Dakrong
Pistoia, Vito
Pitocco, Dario
Pitucha, Monika
Pitzschke, Andrea
Piulachs, Maria Dolors
Piva, Terrence
Plagnol, Vincent
Planchet, Elisabeth
Plass, Katherine E.
Plateroti, Michelina
Platil, Antonin
Pletser, Vladimir
Plotkin, Lilian I.
Pochynyuk, Oleh
Podo, Franca
Poger, David
Pogna, Norberto
Polack, Benoît
Polascik, Thomas J.
Polishchuk, Roman
Pollard, Kenneth M
Pollmann, Katrin
Poloso, Neil
Polyak, Steven
Pompili, Maurizio
Pongsawasdia, Piamsook
Ponton, Fleur
Pook, Mark A.
Poole, David C.
Popanda, Odilia
Poppenberger, Brigitte
Porat, Ron
Porrello, Enzo R.
Porter, Alan L.
Porter, Karen E.
Portero, Manuel
Porteus, Matthew
Post, M.J.
Pothof, Joris
Potumarthi, Ravichandra
Poulsen, Michael
Pourcel, Christine Pourcel
Poyner, David
Prado, Alberto
Praisler, Mirela
Prassl, Ruth
Prati, Fabio
Prato, Mauro
Pratt, Scott L.
Predescu, S.
Preiss, Jack
Prézeau, Laurent
Price, Peter M.
Pridgeon, Julia W.
Prigent-Tessier, Anne
Prince, Roger C.
Princy, Adline
Prinetti, Alessandro
Pringault, O.
Pritchard, Kathleeni
Pritsch, Karin
Proestos, Charalampos
Prokopovich, Polina
Prokunina-Olsson, Ludmila
Proschak, Eugen
Prosser, R. Scott
Prousis, Kyriakos
Provan, Jim
Provenzano, Maurizio
Prudovsky, Igor
Pruitt, Kevin
Psomas, George
Puigdomènech, Pere
Pulliero, Alessandra
Puschenreiter, Markus
Qian, Jiang
Qian, Zhi Rong
Qin, Feng
Qu, Liang-Hu
Quadri, Sadiqa K.
Quaglino, D.
Quan, Taihao
Quaroni, Luca
Que, Long
Quigley, Eamon
Quiroz Figueroa, Francisco
Raamsdonk, Jeremy Van
Rabara, Roel C.
Radchuk, Volodymyr
Radić, Zoran
Radziuk, Darya V.
Raeppel, Stéphane
Rafii, Fatemeh
Rafter, M.A.
Raghunath, Michael
Ragoussis, Jiannis
Rahaman, S. Ohidar
Rahaus, Markus
Rahn, H.
Raivich, Gennadij
Rajala, R.V.S.
Rajamani, Sathish
Rajasekaran, N.S.
Rajasekaran, Sigrid A
Raldúa, Demetrio
Ralph, Stephen J.
Ramachandran, Srinivasan
Ramalho, Teodorico
Raman, Arjun V.
Ramaswamy, Kalyanasundaram
Ramírez-Expósito, María Jesús
Ramji, Dipak P.
Ramos, Sonia
Ramsden, Lawrence
Ramstedt, Madeleine
Randall, Stephen K.
Ranganathan, Sarangarajan
Ranque, Stéphane
Rantamäki, Tomi
Rao, Veena N.
Rappolt, Michael
Rashev, S.
Rassaf, Tienush
Rastelli, Giulio
Rastogui, Gurdeep
Rasul, Azhar
Rath, Thomas
Raub, Chris
Raucci, Mg
Rausell, Carolina
Rawel, H.M.
Re, F.
Recchi, Chiara
Reddy, Krishna
Reddy, P. Hemachandra
Reddy, Umesh K.
Reeve, Amy
Reeves, Helen
Rehmann, Holger
Reich, Kimberly A.
Reich, Reuven
Reichelt, Michael
Rein V, Ulijn
Reiser, Peter J.
Reisman, David
Reiss, Rebecca A.
Renaut, SÉbastien
Renner, Matthias
Renner, Wilfried
Rentsch, Cyrill
Reshetnyak, Yana K.
Revalee, Joel
Reventós, Jaume
Rey, Alejandro D.
Reyes, Fernando
Reynisson, Jóhannes
Reynolds, Nick
Rezzani, Rita
Ribas Fortuny, Judit
Ribeiro, Fernando
Ribeiro, Joaquim A.
Ricci, Giulia
Ricci, V.
Ricciardelli, Carmela
Rice, David
Rice, Scott
Rich, Jeremy N.
Richardson, Alan
Richardson, Christopher
Ridolfi, Elisa
Ridzuan, Mohd A. Mohd
Riello, Pietro
Riemer, Jan
Riley, Brigit E.
Rim, Kyung Taek
Rinaldi, Carlos
Rini, Brian I.
Ris-Stalpers, Carrie
Risk, Janet
Risling, Mårten
Risuleo, G.
Ritter, Helmut
Rittié, Laure
Rius, Francesc Xavier
Rivara, Silvia
Rivas, Ana
Rivera, Jennifer
Rizzo, M.T.
Rizzuti, Bruno
Roach, Melissa
Roberg, Karin
Robert, Jacques
Roberts, David D.
Roberts, Julie
Robin, Charles
Robins, Diane M.
Robson, Craig N.
Rocha, Sonia
Rocheleau, Jonathan V.
Rockwell, Patricia
Roderburg, Christoph
Rodrigues, Clara F.
Rodríguez-Alvarez, José
Rodriguez-Casado, Arantxa
Rodriguez-Lafrasse, Claire
Rodriguez-Navarro, A.
Rodríguez, Ana B.
Rodriguez, Carlos
Roduit, R.
Roelofsen, Han
Roesler, Rafael
Roesner, Lennart M.
Rogenhofer, Sebastian
Rogers, James V.
Roidl, Andreas
Roks, Anton J.M.
Rolletschek, H.
Rollinger, Judith M.
Romano, Rose-anne
Romanowska, Elżbieta
Romero, M.J.
Rommelaere, Guillaume
Ronaldson, Patrick T.
Ronca, Roberto
Rondanelli, Mariangela
Rong, Jianhui
Roos, D.E.
Roos, Raymond P.
Ros, Maria A.
Rosano, Camillo
Rosato, Adriana E.
Rosen, Kirill V.
Rosenzweig, Derek
Rosette, Jean De La
Røsjø, Helge
Rosner, Mordechai
Rossi, H.L.
Rossi, Simona
Rossignol, Daniel
Rossoll, Wilfried
Rota, Rossella
Rothkamm, Kai
Roubelakis, Maria G
Roumenina, Lubka
Roussos, Peter A.
Roviello, Giovanni N.
Rowley, Christopher
Roy, Denis
Roy, Pranab
Rozalska, Barbara
Rubiales, Diego
Rubin, Jeffrey S.
Rubin, Robert L.
Rubio-Cabetas, M.J.
Rubio, Carlos A
Rubio, Ignacio
Ruby, Edward
Rudlowski, Christian
Rudnev, Alexander V.
Ruffalo, Matthew
Ruiz-Cabello, Jesús
Ruiz-Narváez, Edward A.
Ruiz, Encarnación
Ruocco, Michelina
Rupasinghe, H.P. Vasantha
Rupenthal, Ilva D.
Rusin, Aleksandra
Russell, Scott D.
Russell, Stephen J.
Russell, Steven Thomas
Russo, Antonio
Russo, Matteo A.
Rustin, Pierre
Ruszkiewicz, Andrew
Rutkowski, Joseph
Rutkowski, Thomas
Rüttiger, Lukas
Ryter, Stefan W.
Rzeszowska-Wolny, Joanna
Saarinen, Emily
Sabat, Artur
Sabri, Abdel Karim
Sacan, Ahmet
Sacco, Rodolfo
Sachs, Frederick
Sackett, Dan
Sada, Kazuki
Sadee, Wolfgang
Sadok, Walid
Saed, Ghassan M.
Saghatchian, Mahasti
Saha, Gopesh C.
Saha, Sourav
Sahani, Dushyant V.
Saielli, Giacomo
Saikumar, Pothana
Saito, Akira
Saito, Kuniaki
Saito, Reiko
Saito, Takafumi
Saito, Yoshimasa
Saito, Yoshiro
Saitoh, Yasushi
Sajiki, Hironao
Sak, Ali
Sakane, Fumio
Sakwinska, Olga
Sala, Filip A.
Salahinejad, Erfan
Sale, Alessandro
Saleh, Mahmoud A.
Salerno, Marco
Salgado, Jesús
Salgueirino, Veronica
Saliba, Kevin J.
Sallese, Michele
Salmon, Adam
Salta, Maria
Salter, Donald
Saluja, Ashok K.
Saluzzo, Christine
Salzman, David
Samanidou, Victoria
Samimi, Goli
Sampaolesi, Maurilio
Samulski, Jude
Sanada, Hiromi
Sanbonmatsu, Karissa Y.
Sánchez-Barceló, Emilio
Sanchez-Elsner, Tilman
Sánchez-Sánchez, Manuel
Sánchez-Soto, Miguel
Sanchez, Aranzazu
Sanchez, Diego H.
Sancho, Elena
Sancho, Patricia
Sanders, Charles R.
Sandoval, Juan
Sanejouand, Yves-Henri
Sani, Rajesh
Sanmartín, Carmen
Sanoh, Seigo
Santoni, Matteo
Santos-Buelga, Celestino
Santos, Claudio R.
Santos, José L.
Santucci, Annalisa
Sapino, Anna
Sardao, Vilma
Sargan, David R.
Sarrà, M.
Sartore Bianchi, Andrea
Sasaki, Hiroki
Sastre, Juan
Sato, Fumihiko
Sato, Jun
Sato, Masa H.
Sato, Tatsuo
Sato, Yasufumi
Sato, Yutaka
Satoh, Hisashi
Satoh, Shigeru
Satten, Glen
Satyanarayanajois, Seetharama D.
Saunders, Philippa T.K.
Saunders, Thom
Savaskan, Nic E
Savic, J.M.
Saville, Barry J.
Savitch, Leonid
Sawyer, Gregory W.
Sayasneh, Ahmad
Scaman, Christine H.
Scanes, Colin G.
Scarpella, Enrico
Schaefer, Jeremy
Schaefer, Michael
Schäfer, Katrin
Schaper, Fred
Scheckhuber, Christian Q.
Scheibe, Blazej
Schellenberg, Peter
Scheller, Henrik Vibe
Schemmer, Peter
Schena, Francesco P.
Schenck, Carlos
Scherer, Günther
Schernthaner, Gerit-Holger
Schillberg, Stefan
Schinke, Thorsten
Schipani, Ernestina
Schippers, Jos
Schlaepfer, Isabel R
Schleinitz, Dorit
Schlüter, Hartmut
Schmid, Friederike
Schmid, Kara
Schmidt, Alex
Schmidt, Erwin R.
Schmidt, Marius
Schmit, Jeremy
Schmitz, A.
Schmitz, Matthias
Schnabl, Bernd
Schneider, Sallie
Schnier, Paul D.
Schols, Dominique
Scholz, Bettina
Scholz, Michael
Schöpfer, Jutta
Schrauder, Michael G.
Schraufstatter, Ingrid U.
Schreier, A.D.
Schrobback, Karsten
Schulte, Ulrich
Schultz, Elizabeth A.
Schulz, Ekekhard
Schulze-Tanzil, Gundula
Schuman, Thomas
Schwarcz, Robert
Schwarz, Quenten
Schweizer, Frank
Schwertfeger, Kaylee
Scorilas, Andreas
Scorza, Tatiana
Scotognella, Francesco
Scott, H. Larry
Scott, Michelle S.
Searles, Charles D.
Sears, Dorothy D.
Secchiero, Paola
Secord, Angeles Alvarez
Seed, Brian
Seemann, P.
Segal, Anthony W.
Seifert, Gotthard
Seiffert, Sebastian
Seki, Naohiko
Sekino, Masashi
Selan, Laura
Seligman, Paul A.
Seligmann, H.
Sell, Henrike
Seluanov, Andrei
Selvam, Thangaraj
Semaltianos, N.G.
Semenza, Gregg L.
Sempere, Lorenzo
Sen, Suvajit
Sena, Cristina M.
Senbayram, Mehmet
Senchina, David S.
Senet, Patrick
Senna Salerno, Mônica
Seo, Shigemi
Seo, Young-Kwon
Sepulveda, Pilar
Serhan, Charles N.
Serizawa, Takeshi
Serralheiro, Maria Luísa M.
Serrano, L.
Seth, Devanshi
Sette, Claudio
Setter, Tim L.
Sgadò, Paola
Shabala, Sergey
Shabbir, Muhammad
Shah, Ketan
Shah, Parag P.
Shaikh, Zahir A.
Shaikhali, Jehad
Shang, Xiying
Shao, Changshun
Shao, R.
Shaposhnik, Zory
Sharabi, Andrew
Shareck, F.
Sharma, Alok
Sharma, Aruna
Sharma, Jyotsna
Sharma, Mridula
Sharma, Rajendra
Sharman, Edward H.
Sharpe, Paul T.
Shavrukov, Y.
Shaw, Huey-Mei
Shay, Jerry W.
Shen, Chwan-Li
Shen, Jinfeng
Shen, Wei
Shen, Yu
Shen, Yuh-Chiang
Shendruk, T.N.
Sheng, Baiyang
Sheng, Yugang
Sherer, Nathan
Sherman, Mark E.
Shertzer, Howard G.
Shetty, Pavan K.
Sheu, Joen R.
Sheu, Jyh-Horng
Shibasaki, Futoshi
Shih, Ming-Chih
Shikano, Takahito
Shim, Yhong-Hee
Shim, Young Key
Shimada, Masako
Shimizu, Bun-Ichi
Shimizu, Kazuyuki
Shimoda, Hiroshi
Shin, Chan Young
Shinoda, Wataru
Shiota, Masaki
Shirasaka, Norifumi
Shirasawa, K.
Shiratake, Katsuhiro
Shiromani, Priyattam
Shore, Neal
Shoshan, Maria
Shoyama, Yukihiro
Shurin, Michael R.
Shyu, Huey-Wen
Siddle, K.
Sidi, Yechezkel
Sieczkowska, Mirosława Ferens
Siegal, Gene P.
Siegel, Sue Rutherford
Siejka, Agnieszka
Siest, Gérard
Sieuwerts, Anieta M.
Sievers, Claudia
Sikarwar, Anurag
Sikes, Robert A.
Siligardi, Giuliano
Silikas, Nick
Sill, Heinz
Silljé, Herman H.W.
Silva, A.C.
Silva, Amanda
Silva, Liana C.
Silva, Sn
Silvers, Robert
Simal-Gándara, Jesús
Simon, Martin
Simon, O.
Simoncic, Barbara
Simplicio, Paolo
Simpson, Guy
Sinclair, Thomas R.
Sing, Om V.
Singal, Ashwani
Singh, Ishwar S.
Singh, Kumud K.
Singh, Neenu
Singh, Pomila
Singh, S.K.
Singhal, Naresh
Sinha-Hikim, Amiya P.
Siniscalco, Dario
Sintim, Herman O.
Sirangelo, Ivana
Siwek, Agata
Skaper, Stephen D.
Skeldal, Sune
Skelding, Kathryn A.
Skipworth, R.J.E.
Skovgaard, Kerstin
Skwarlo-Sonta, K.
Skwarzec, Bogdan
Sliva, Daniel
Sliz, Piotr
Sloane, Bonnie
Slominski, Andrzej
Slomovitz, Brian M.
Slovin, Susan
Slyvka, Yuriy
Smalheiser, Neil R.
Smart, Lawrence B.
Smith, Bernard Rees
Smith, Caroline
Smith, Ed
Smith, Judith A.
Smith, Pamela A.
Smith, Wanli
Smyth, Redmond
Soares-Silva, Isabel João
Sobrio, F.
Söderhäll, Irene
Soengas, Raquel
Sohn, Jeongwon
Sokol, Pamela A.
Sokolow, Sophie
Solda, Giulia
Soleño, Jimena
Sollars, Vincent E.
Sommer, Sebastian-Patrick
Somoza, Gustavo
Son, Deok-Soo
Sone, Hideko
Song, Bang-Ho
Song, Jianxun
Song, Shuhui
Song, Yiqing
Sonkoly, Enikö
Sonkusare, Swapnil
Sonnino, Sandro
Sonoda, Akinaga
Soreq, Hermona
Soroceanu, Liliana
Soto, Maria
Souglakos, J.
Soulé, Ezequiel R.
Soulère, Laurent
Soulimane, Tewfik
Soultos, Nikolaos
Souza, Marcos
Sowers, James R.
Spagnoli, Fm
Spampinato, Santi
Spanier, Britta
Spanos, Kostas
Spasojevic, Ivan
Spee, Bart
Speeckaert, Marijn
Speicher, David J
Sperry, Ann O.
Spivey, Alan C.
Sreekumar, Parameswaran G.
Sreeram, K.J.
Sridhar, Jayalakshmi
Sridharan, Vijayalakshmi
Srivastava, Anup
St. Louis, Erik K.
Staels, Bart
Stahelin, Robert V.
Stahl, Wilhelm
Stahl, Wolfgang
Staibano, Stefania
Stam, Ronald W.
Stamova, Boryana S.
Stankovic, Tanja
Stather, P.W.
Steen, Judith A.J.
Steenbergen, Renske D.M.
Stefanidou, Maria E.
Stefanis, Leonidas
Stefano, Cassanelli
Stefano, Cesco
Stehle, Jörg H.
Stein, Cy A.
Stein, Thor
Steinberger, Peter
Steinestel, Konrad
Stelinski, Lucasz
Stenglein, Sebastian
Stepensky, David
Stern, Michael
Stewart, Gavin
Stewart, Grant S.
Stiborova, Marie
Stieger, Bruno
Stiles, Bangyan L.
Stobiecki, Maciej
Stocker, Claire J.
Stojanovski, Diana
Storey, Douglas G.
Stotz, Michael
Strauss, Olaf
Strauß, Sarah
Streit, W.R.
Stricker, Sigmar
Stroe, Izabela
Strom, Jakob O.
Strömvik, Martina V.
Stronach, Euan
Stuckey, Jeanne A.
Stuetz, Evamaria
Stunes, Astrid Kamilla
Stuppia, Liborio
Stutzmann, Grace E.
Su, Maureen A.
Su, Tin Tin
Su, Tsung-Ping
Subbaiah, Papasani Venkata
Subramanian, Senthil
Subudhi, Prasanta K.
Suenaga, Hikaru
Sugasawa, Kaoru
Sugawara, Akira
Suggs, Laura J.
Sugihara, Takashi
Sugimoto, Hiroyuki
Sugimoto, Mikio
Sugimura, Haruhiko
Sugimura, Kazuhisa
Sugiyama, Seigo
Sugiyama, T.
Sugumaran, Manickam
Suh, Yoo-Hun
Sumi, Shoichiro
Sun, Grace
Sun, Hongmin
Sun, Jessica D.
Sun, Jin
Sun, Shaogang
Sundelin, Thomas
Sung, Hak-Joon
Sunkara, Lakshmi
Suo, Satoshi
Supiot, Stéphane
Surette, Marc E.
Sutela, Suvi
Suzuki, Hiroetsu
Suzuki, Hiromu
Suzuki, Hiroyoshi
Suzuki, Keiji
Suzuki, Satoru
Suzuki, Tadashi
Suzuki, Takayoshi
Suzuki, Takuya
Sydow, Carsten
Sylvestre, Michel
Symonds, Vaughan
Szalai, G.
Szászi, Katalin
Szczepanik, Beata
Szczubiałka, Krzysztof
Sze, Newman
Szeberényi, József
Szmidt, Alfred E.
Szweykowska-Kulinska, Zofia
Tadege, Million
Taglialatela-Scafati, Orazio
Taglialatela, Maurizio
Taglietti, Angelo
Tago, Megumi
Tai, Sorgan
Tajbakhsh, J.
Tajmir-Riahi, H.A.
Takabayashi, Junji
Takada, Toshinori
Takahashi, Hideyuki
Takami, Seiichi
Takanishi, Yoichi
Takano, Kazufumi
Takayama, Fusako
Takei, Yoshiyuki
Takemura, Genzou
Takeshita, Fumitaka
Takeya, Kosuke
Takumi, Shigeo
Talbert, Joey N.
Tamanoi, Fuyuhiko
Tampieri, Anna
Tan, Aik Choon
Tan, Dun-Xian
Tan, Kee W.
Tan, Zhenquan
Tanabe, Shihori
Tanaka, Hiroaki
Tanaka, Hiroshi
Tanaka, Hitoshi
Tanaka, Ryouichi
Tanaka, Takuji
Tanaka, Tsuyoshi
Tandon, Vishnu K.
Tang, Beibei
Tang, Bo
Tang, Bor Luen
Tang, Chih-Hsin
Tang, Chih-Yung
Tang, Hua
Tanigawa, Tetsuya
Taniguchi, Mitsutaka
Tanimoto, A.
Tanner, John J.
Tanonaka, Kouichi
Tantry, Mudasir A.
Tao, Wendong
Tao, Ya-Xiong
Taponen, Suvi
Tarasov, Sergey A.
Taratula, Oleh
Tarozzi, Andrea
Tartoura, Kamel A.H.
Tarver, James E.
Tarzami, Sima T.
Tasciotti, Ennio
Tassa, Jean-Pierre
Tassanakajon, Anchalee
Tato, José Vázquez
Taverna, Daniela
Tavridou, Anna
Tawil, Bill J.
Tay, Samuel Sam Wah
Taylor, Maureen E.
Taylor, T. Matthew
Te Pas, Marinus F.W.
Teismann, Peter
Teixeira, Natércia
Templeton, Douglas M.
Tenreiro Machado, J.A.
Terauchi, Ryohei
Terazzi, E.
Terenzi, Hernán F.
Tero, Ryugo
Terracciano, Daniela
Terracciano, Rosa
Terrier, Nancy
Terry, Kathryn L.
Teske, Jennifer A.
Thalhammer, G.
Thathiah, Amantha
Theise, Neil D.
Theiss, Carsten
Thevananther, Sundararajah
Thévenod, Frank
Thi, Mia Mia
Thierry, Alain R.
Thijssen, Dick H.J.
Thilmony, Roger
Thippeswamy, Thimmasettapp
Thomas, Andrew G.
Thornton, Geoff
Thumser, Alfred E.
Tian, Jun
Ticozzi, Nicola
Tien, Hwei-Fang
Tikkanen, Ritva
Tilkin-Mariamé, Anne-Françoise
Timm, Janne
Timsit, Youri
Ting, Kang
Tinto, Nadia
Titorenko, Vladimir I.
Tjan-Heijnen, Vivianne C.G.
Tobin, Gunnar
Tocco, Ilaria
Todaka, Daisuke
Todorova, Nevena
Tognini, Paola
Tohge, Takayuki
Tohno, Masanori
Toimil, Paula
Tolar, J.
Tomasetto, Catherine
Tomasi, Nicola
Tomatis, Maura
Tong, Sheng
Tonomura, Y.
Too, Heng-Phon
Topisirovic, Ivan
Torlakovic, Emina E.
Török, Marianna
Torre, Vincent
Torres, Anna
Torres, Cesar
Torres, Lani
Torrisi, Maria Rosaria
Tosi, Giovanni
Tota, Bruno
Touboul, David
Touil-Boukoffa, Chafia
Touitou, Yvan
Toyofuku, Masanori
Toyooka, Tatsushi
Tozzoli, Renato
Trabert, Britton
Trakht, Ilya
Tran, Nhiem
Tranbarger, Timothy
Treeck, Oliver
Trendelenburg, George
Tress, Michael
Treussart, François
Trevaskis, Ben
Trijatmiko, Kurniawan Rudi
Trincavelli, Maria Letizia
Tripathi, Durga Nand
Tripathi, Prateek
Trivedi, Pankaj
Trobacher, Christopher
Troev, Kolio
Trojano, Maria
Trompeter, Hans-Ingo
Trost, Paolo
Trougakos, Ioannis P.
Trouillas, Patrick
Trouvelot, Sophie
Trovato, G.M.
Trudel, Dominique
Trueb, Beat
Tsai, Fuu-Jen
Tsai, Hui-Hsu Gavin
Tsai, Ming-Shan
Tsai, Po-Jung
Tsang, Michael
Tsao, Yeou-Ping
Tseng, W.L.
Tsiotra, Panayoula C.
Tsoporis, James N.
Tsuda, Hitoshi
Tsuji, Yoshiaki
Tsukamoto, Hidekazu
Tsumura, Yoshihiko
Tsutsumi, Yasuo
Tucci, Paola
Tuffery, Pierre
Tullu, Abebe
Tunster, S.J.
Turhan, Ali G.
Turner, Anthony J.
Turner, David
Turner, Neil A.
Tuzimski, Tomasz
Tyagi, Suresh C.
Tyedmers, Jens
Ubukata, Takashi
Uchinami, Hiroshi
Uddin, Muhammad Jasim
Ueda, Koji
Ueda, Seiji
Uehara, Tohru
Ueki, Tatsuya
Uen, Yih-Huei
Ueng, Yune-Fang
Ugalde, Unai
Uhde-Stone, C.
Uhlmann, Dirk
Ulmer, Tobias S.
Umehara, Hisanori
Unfer, V.
Uozumi, Nobuyuki
Urabe, Hirokazu
Urade, Reiko
Uray, Karen L.
Urbanc, B.
Urbany, Claude
Urushitani, Makoto
Urzì, Clara E.
Usta, Berk
Vainio, Eeva J.
Vaira, Valentina
Valdés, Ana Elisa
Valdez, Benigno C.
Valencia, Antonio
Valencia, C. Alexander
Valentová, Kateřina
Valero, M.
Valix, Marjorie
Vallejo, Yli Remo
Van Aerschot, Arthur
Van Berkel, Willem
Van Cutsem, Pierre
Van Den Bogaard, Ellen H.
Van den Broeck, Hetty
Van Der Burgt, Yuri E.M.
Van der Kamp, Marc W.
Van Der Kwas, Theo H.
Van Doorn, Arjen
Van Doorn, W.G.
Van Endert, Peter
Van Gent, Dik C.
Van Gijsegem, Frédérique
Van Griensven, Leo J.L.D.
Van Ijzendoorn, Sven C.D.
Van Laere, S.J.
Van Leeuwen, Thomas
Van Nieuw Amerongen, Geerten
Van Rijn, Richard M.
Van Zonneveld, Anton Jan
Vandeputte, Olivier M.
Vander Griend, Donald J.
Vanderlinde, Elizabeth
Vaquero, M. Pilar
Vargas, Félix
Vargas, W.A.
Vargo-Gogola, Tracy
Vasko, Vasyl
Vasquez, Karen
Vassella, Erik
Vayssade, Muriel
Vaz, Roy J.
Vazquez, M. Dolores
Vecchione, Carmine
Veintemillas-Verdaguer, Sabino
Veit-Haibach, Patrick
Velazquez-Campoy, Adrian
Venable, Mark E.
Venault, Antoine
Venditti, Alessandro
Venier, Paola
Ventura, M. Rita
Ventura, Salvador
Venturi, Vittorio
Veracini, Carlo Alberto
Verburg-Van Kemenade, B.M.L.
Vercellino, Tony
Verchot-Lubicz, Jeanmarie
Verderio, Claudia
Vergoten, Gérard
Verhoeyen, Els
Verlinden, Heleen
Vermeulen, Louis
Vernoud, Vanessa
Veronesi, Bellina
Verri, Tiziano
Vetter, Stefan W.
Vetterkind, Susanne
Vetvicka, Vaclav
Vianello, Fabio
Viani, Gustavo Arruda
Vicentini, Lucia M.
Vieira, A.
Vieira, C.P.
Vieira, Filipe G.
Vijayan, Perumal
Viladomat, Francesc
Villa, Federica
Villares, Ana
Villari, Valentina
Villers, Arnauld
Vimalanathan, Selvarani
Vinall, Ruth
Viñals, Francesc
Vinceti, Marco
Vinciguerra, Manlio
vindigni, vincenzo
Vingtdeux, Valérie
Vinken, Mathieu
Viola, G.
Virdi, Amarjit S.
Virgilli, Fabio
Visavadiya, Nishant P.
Visioli, Francesco
Vitale, Giovanni
Vitantonio, Pantaleo
Vitetta, Luis
Vittal, Ragini
Vlachonasios, K.E.
Vlahou, Antonia
Voelkel-Johnson, Christina
Vogel, Kristine S.
Volinia, Stefano
Volmer, Dietrich
Vomastek, Tomas
Voncken, Jan Willem
Vorachek, William R.
Vorsa, Nicholi
Vorstenbosch, Joshua
Vovk, Andriy I.
Vozzi, Giovanni
Vrailas-Mortimer, Alysia
Vrček, Ivana Vinković
Vreede, Jocelyne
Vucenik, Ivana
Wach, Sven
Wadhwa, Renu
Waeber, Christian
Wagley, Yadav
Wagner, Brent
Wagner, Deborah S.
Wahlestedt, Claes
Wakagi, T.
Wakeel, Abdul
Walde, Peter
Walenkamp, Annemiek
Walorczyk, Stanisław
Walsh, John P.
Walsh, William R.
Walters, D.R.
Walton, J.C.
Wan, Fengyi
Wan, Qi
Wan, Yinsheng
Wanasundara, Janitha
Wang, Bo-Cheng
Wang, Cheng-Hsu
Wang, Chenguang
Wang, Chih-Yu
Wang, Dongping
Wang, Guocheng
Wang, Jaw-Yuan
Wang, Jianping
Wang, Jiemei
Wang, Jinghua
Wang, Jun
Wang, Kai
Wang, Kevin
Wang, Le
Wang, Leo D.
Wang, Li
Wang, Meng-Jiy
Wang, P.
Wang, Peng George
Wang, Qi-En
Wang, Qiaomei
Wang, Qun
Wang, Run
Wang, Shusheng
Wang, Songhu
Wang, Wanxin
Wang, Wei-lin Winnie
Wang, Xiajou
Wang, Xinkun
Wang, Yanwei
Wang, Yi-Ching
Wang, Yi-Hong
Wang, Ying
Wang, Yongjun
Wang, Yu
Wang, Yun
Wang, Zhou
Wanke, Dierk
Wanker, Erich
Wansink, Derick G.
Ward, Jeanine
Waris, Gulam
Watanabe, Kazuhito
Watarai, Hitoshi
Watters, Michael K.
Wattraint, Olivier
Weatherall, David
Weber, Franz E.
Ween, Miranda
Wei, Alexander
Wei, Ning
Wei, Qingrong
Wei, Robert
Wei, Ting
Weichhart, Thomas
Weickert, Martin O.
Weidlich, Sandy
Weijers, Rob N.M.
Weinberg, Annelie M.
Weiner, Steve
Weinhäusel, Andreas
Weiser, Douglas C.
Weiss, Louis M.
Weissmann, Norbert
Welch, Martin
Weltzien, Finn-Arne
Wendel, A.
Weng, Alexander
Weng, Ching-Feng
Wenzel, Daniela
Wesseling, Jelle
Westbroek, Wendy
Westbury, C.B.
Westenfelder, Christof
Westerhout, Ellen M.
Westmark, Cara J.
Weston, Leslie A.
Westwell, Andrew D.
Wheeler, Ward C.
White, Rosemary
Whiteford, James R.
Whitelegge, Julian P.
Whitelock, John
Whitnall, Mark H.
Whittell, George
Widlak, Piotr
Wiechmann, Allan
Wiedwald, Ulf
Wiegand, Claudia
Wiemer, Erik A.C.
Wienkoop, Stefanie
Wigdahl, Brian
Wigren, Maria
Wilc0x, Christopher S.
Wilde, P.J.
Wiles, Charlotte
Wilgus, T.A.
Wilhelm, Claire
Wilkins, Ruth
Willand, Nicolas
Williams, Dustin L.
Williams, S.T.
Willis, Monte S.
Wilson, Brenda
Wilson, D.B.
Wilson, Elizabeth
Wilson, Iain
Wilson, Joyce A.
Winans, Stephen C.
Windsor, L. Jack
Winter, Jeannette
Wirths, Oliver
Wischhusen, Joerg
Wislet-Gendebien, S.
Wisztorsk, Maxence
Witte, Wolfgang
Wittmann, Jürgen
Wittung-Stafshede, Pernilla
Wnek, Gary E.
Wojcik, Andrzej
Wolf, Jennifer Moriatis
Wong, Alice S.T.
Wong, Chun-Ming
Wong, Eric A
Wong, K.-K.
Wong, Kenneth K.Y.
Wong, Victor W.
Wong, W.S. Fred
Woo, Se Joon
Wood, Fiona
Wood, Janet M.
Wozniak, Magdalena B.
Wragg, David S.
Wren, Anthony
Wright, Gary
Wu, Baojian
Wu, Cheng-Hsun
Wu, Hua-Lin
Wu, Jaw-Ching
Wu, Jiahe
Wu, Jian
Wu, Jiunn-Tzong
Wu, Junfang
Wu, Keqiang
Wu, Lianfeng
Wu, M.
Wu, Mousheng
Wu, Shiyong
Wuertz, Karin
Wurtz, Peter
Wyse, Angela T.S.
Xavier, Karina B.
Xia, Tian
Xia, Ying
Xian, Ming
Xiao, Li
Xie, C.-H.
Xie, Guanhua
Xie, Jun-Xia
Ximenes, Valdecir F.
Xin, Wei
Xiong, Ye
Xiong, Yuquan
Xu, Baoshan
Xu, Jian
Xu, Li-Hong
Xu, Xiaochun
Xu, Yan
Xu, Yiru
Xu, Zheng-Hong
Yadegari, Ramin
Yager, James
Yakovlev, Vladislav
Yamada, Sohsuke
Yamagishi, M.
Yamaguchi, Yoshiki
Yamakawa-Kobayashi, Kimiko
Yamamoto, Kotaro T.
Yamamoto, Megumi
Yamamoto, Takakazu
Yamaoka, Masuo
Yang, Bo
Yang, Chia-Ron
Yang, Chuen-Mao
Yang, Decheng
Yang, Deng-Ke
Yang, Guang-Dong
Yang, Guangdong
Yang, Kwangmo
Yang, Qiwei
Yang, Rong Sen
Yang, Shang-Hsun
Yang, Shiping
Yang, Shuhua
Yang, Shun-Fa
Yang, Wanling
Yang, Xiang-Jiao
Yang, Xiaojing
Yang, Xiuqing
Yang, Zhijun
Yano, Shozo
Yao, Youli
Yasuda, Hiroyuki
Yasuda, Jun
Yates, Matthew Z.
Ye, Keqiang
Yeh, Hung-Yueh
Yeh, I-Tien
Yenari, Midori A.
Yepiz-Plascencia, Gloria
Yeudall, W. Andrew
Yeung, Kam C.
Yeung, Sai-Ching Jim
Yin, Huabing
Yin, Peng
Yip, Christopher M.
Yli-Mattila, Tapani
Yokosuka, Makoto
Yokoyama, K.
Yokoyama, Kazunari K.
Yokoyama, Shinji
Yokoyama, Takashi
Yokoyama, Yoshihito
Yomo, Shoji
Yoneyama, Koichi
Yonezawa, Tomo
Yoo, Yeong-Min
Yoo, Young Hyun
Yoon, Seog-Young
Yoshiba, Sayaka
Yoshida, Hiderou
Yoshida, Hiroyuki
Yoshida, Yukihiro
Yoshiji, Hitoshi Yoshiji
Yoshikawa, Kenichi
Yoshimoto, Makoto
Yoshimoto, Takayuki
Young, Chiu-Chung
Young, Robert S.
Yu, Cheng-Ping
Yu, Diqiu
Yu, Il Je
Yu, Lijian
Yu, Mengxiao
Yu, Tien-Shin
Yu, Yan Ping
Yu, Zhanyang
Yuan, Jiayin
Yue, Genhua
Yue, Patrick Ying Kit
Yun, Hyungdon
Yun, Yeoheung
Yung, Susan
Yura, Yoshiaki
Yuste, Victor J.
Zacarias, L.
Zaccardelli, Massimo
Zacharof, Myrto-Panagiota
Zafari, A. Maziar
Zagdanska, Barbara
Zagrovic, Bojan
Zakas, Christina
Zakharova, Lucia
Zaman, Farasat
Zaman, Khalequz
Zamaratskaia, Galia
Zang, Qingda (Dan)
Zarogoulidis, Paul
Zarrine-Afsar, Arash
Zarzycki, Paweł K.
Zaslau, S.
Zaugg, Michael
Zawilska, Jolanta B.
Zechmann, Bernd
Zefirov, Nikolay
Zeidler, Reinhard
Zeillinger, Robert
Zelikoff, Judith
Zell, Roland
Zempleni, Janos
Zeng, Taofang
Zeng, Xiankun
Zetterberg, Fredrik
Zhan, Aibin
Zhang, Dapeng
Zhang, Dawei
Zhang, Hanrui
Zhang, Jie
Zhang, Jinsong
Zhang, Junran
Zhang, Li
Zhang, Li Xin
Zhang, Miao
Zhang, Qunzhou
Zhang, Wen
Zhang, Wenheng
Zhang, Wenqing
Zhang, Xiaonong
Zhang, Yiqiang
Zhang, Yu
Zhang, Yunxia
Zhao, Chunfeng
Zhao, Lixia
Zhao, Nan
Zhao, Qiang
Zhao, Xiaojun
Zhao, Yutong
Zhao, Zhun
Zheng, G.
Zheng, Heping
Zheng, Jie
Zheng, Shu-Sen
Zheng, Wenguang
Zheng, Xiangzhong
Zhou, Beiyan
Zhou, Dan
Zhou, Huan
Zhou, Huiqing
Zhou, Huitong
Zhou, Qingxiang
Zhou, Shuanhu
Zhou, Xuan-Wei
Zhou, Zhuan
Zhu, Changfu
Zhu, Heng
Zhu, Lixin
Zhu, Qian-Hao
Zhuang, Zhihao
Ziebell, Jenna M.
Zielske, Steven P.
Zilbauer, Matthias
Zimmer, Bastian
Zink, M.
Zlotos, Darius P.
Zmijewski, Jaroslaw W.
Zolak, Jason S.
Zöller, Margot
Zollo, Massimo
Zou, Hejian
Zou, Jun
Zrobek-Sokolnik, A.
Zúñiga-Pflücker, Juan Carlos
Zunino, Franco
Zuppinger, Christian

